# IL-1R-IRAKM-Slc25a1 signaling axis reprograms lipogenesis in adipocytes to promote diet-induced obesity in mice

**DOI:** 10.1038/s41467-022-30470-w

**Published:** 2022-05-18

**Authors:** Weiwei Liu, Hao Zhou, Han Wang, Quanri Zhang, Renliang Zhang, Belinda Willard, Caini Liu, Zizhen Kang, Xiao Li, Xiaoxia Li

**Affiliations:** 1 Department of Inflammation and Immunity, Lerner Research Institute, Cleveland Clinic, Cleveland, OH 44106 USA; 2 Center for RNA Science and Therapeutics, School of Medicine, Case Western Reserve University, Cleveland, OH 44106 USA; 3 Department of Biochemistry, School of Medicine, Case Western Reserve University, Cleveland, OH 44106 USA; 4 Department of Computer and Data Sciences, School of Engineering, Case Western Reserve University, Cleveland, OH 44106 USA; 5 Proteomics and Metabolomics Core, Lerner Research Institute, Cleveland Clinic, Cleveland, OH 44106 USA; 6 Department of Pathology, Carver College of Medicine, University of Iowa, Iowa City, IA 52242 USA; 7 Present Address: Division of Transplant Surgery, Department of Surgery, Brigham and Women’s Hospital, Harvard Medical School, Boston, MA 02115 USA

**Keywords:** Chronic inflammation, Kinases, Obesity

## Abstract

Toll-like receptors/Interleukin-1 receptor signaling plays an important role in high-fat diet-induced adipose tissue dysfunction contributing to obesity-associated metabolic syndromes. Here, we show an unconventional IL-1R-IRAKM-Slc25a1 signaling axis in adipocytes that reprograms ﻿lipogenesis to promote diet-induced obesity. Adipocyte-specific deficiency of IRAKM reduces high-fat diet-induced body weight gain, increases whole body energy expenditure and improves insulin resistance, associated with decreased lipid accumulation and adipocyte cell sizes. IL-1β stimulation induces the translocation of IRAKM Myddosome to mitochondria to promote de novo lipogenesis in adipocytes. Mechanistically, IRAKM interacts with and phosphorylates mitochondrial citrate carrier Slc25a1 to promote IL-1β-induced mitochondrial citrate transport to cytosol and de novo lipogenesis. ﻿Moreover, IRAKM-Slc25a1 axis mediates IL-1β induced Pgc1a ﻿acetylation to regulate thermogenic gene expression in adipocytes. IRAKM kinase-inactivation also attenuates high-fat diet-induced obesity. ﻿Taken together, our study suggests that the IL-1R-IRAKM-Slc25a1 signaling axis tightly links inflammation and adipocyte metabolism, indicating a potential therapeutic target for obesity.

## Abstract (web summary)

TLRs/IL-1R signaling play a role in high-fat diet induced adipose tissue dysfunction and obesity-associated metabolic syndrome. Here the authors report that adipocyte-specific deficiency of IL-1 Receptor-Associated Kinase M (IRAKM) ameliorates diet induced obesity, potentially via the mitochondrial citrate carrier Slc25a1 and reduced de novo lipogenesis.

## Introduction

Obesity is a major risk factor for insulin resistance, type 2 diabetes (T2D), and metabolic syndrome^1–4^. When energy intake chronically exceeds energy expenditure, positive energy balance is sustained, resulting in weight gain, fat accumulation in adipose tissues, and obesity^5,6^. ﻿Notably, low-grade inflammation in adipose tissues is strongly associated with excess body fat mass and ﻿insulin resistance^7,8^. High-fat diet (HFD) induces the accumulation of endogenous TLR ligands [free fatty acids (FFAs), oxidized low-density lipoprotein (ox-LDL)], and inflammatory cytokines such as Interleukin-1 (IL-1)^9–12^. Emerging evidences indicate that Toll-like receptor (TLR)/interleukin-1 receptor (IL-1R) signaling plays an important role in HFD-induced adipose tissue dysfunction^13–15^.

TLR/IL-1R transduce signals through the adapter molecule MyD88 and IL-1R-associated kinase (IRAK) family members, including IRAK1, IRAK2, IRAKM, and IRAK4^16^. TLR/IL-1R activation leads to the formation of Myddosome complexes (MyD88-IRAK4-IRAK1; MyD88-IRAK4-IRAK2; MyD88-IRAK4-IRAKM) required for NF-κB activation and the production of inflammatory cytokines^17,18^. We previously reported that TLR/IL-1R adaptor MyD88 deficiency in myeloid cells prevented macrophage recruitment to adipose tissue and their switch to an M1-like phenotype, accompanied by substantially reduced diet-induced systemic inflammation and insulin resistance^19^. More recently, we reported that IRAK2 Myddosome is translocated into the mitochondria where IRAK2 interacts with PHB and OPA1 to suppress OxPhos and fatty acid β-oxidation, implicating TLR/IL-1R signaling in adipocyte metabolism^20^. However, it remains unclear whether and how the other IRAK Myddosomes participate in unconventional TLR/IL-1R signaling in adipocytes. More importantly, how the impact of TLR/IL-1R signaling on adipocyte metabolism contributes to positive energy balance, fat accumulation, and obesity.

Adipocyte hypertrophy (enlargement due to lipid accumulation) was tightly correlated with dramatic increase of fat mass and increased systemic insulin resistance in obesity^21,22^. Moreover, the ability of adipocytes to synthesize de novo fatty acids from glucose contributes to lipid storage during adipocyte hypertrophy^23^. ﻿Cytosolic metabolism of citrate to acetyl-coenzyme A (acetyl-CoA) is important for de novo fatty acid synthesis (FAS)^24^. ﻿Citrate produced in the citric acid cycle (TCA cycle) inside mitochondria can be exported from mitochondria to cytosol via the mitochondrial citrate carrier solute carrier family 25 member 1 (Slc25a1) ﻿which regulates the cytoplasmic and mitochondrial pools of citrate^25^. Notably, recent studies showed that TLR signaling driven FAS pathway promotes dendritic cell and macrophage activation^26,27^. However, the molecular mechanisms for how TLR/IL-1R signaling reprograms adipocytes metabolism, especially FAS and the impact on obesity-associated metabolic syndrome remains unclear.

Here we report that adipocyte-specific deficiency of IRAKM reduces high-fat diet-induced body weight gain, increases whole-body energy expenditure, and improves insulin resistance. We showed that IL-1β stimulation induces the translocation of IRAKM Myddosome to mitochondria to promote de novo fatty acid synthesis in adipocytes. Mechanistically, IRAKM interacts with mitochondrial citrate carrier Slc25a1 to promote IL-1β-induced mitochondrial citrate transport to cytosol. In vitro studies revealed that Slc25a1 is a direct substrate of IRAKM. Furthermore, Slc25a1 phosphorylation induced by IRAKM kinase activity is required for its citrate transport activity and de novo fatty acid synthesis upon IL-1β stimulation. ﻿Moreover, IL-1β induces Pgc1a ﻿acetylation via IRAKM-Slc25a1 axis to regulate thermogenic gene expression in adipocytes. Accordingly, IRAKM kinase-inactive knock-in attenuates HFD-induced obesity. ﻿Taken together, these data suggest an unexpected role for IRAKM in reprogramming fatty acid synthesis in adipocytes, contributing to obesity-associated adipose tissue dysfunction.

## Results

### Adipocyte-specific IRAKM deficiency attenuates HFD-induced obesity

**Fig. 1:**
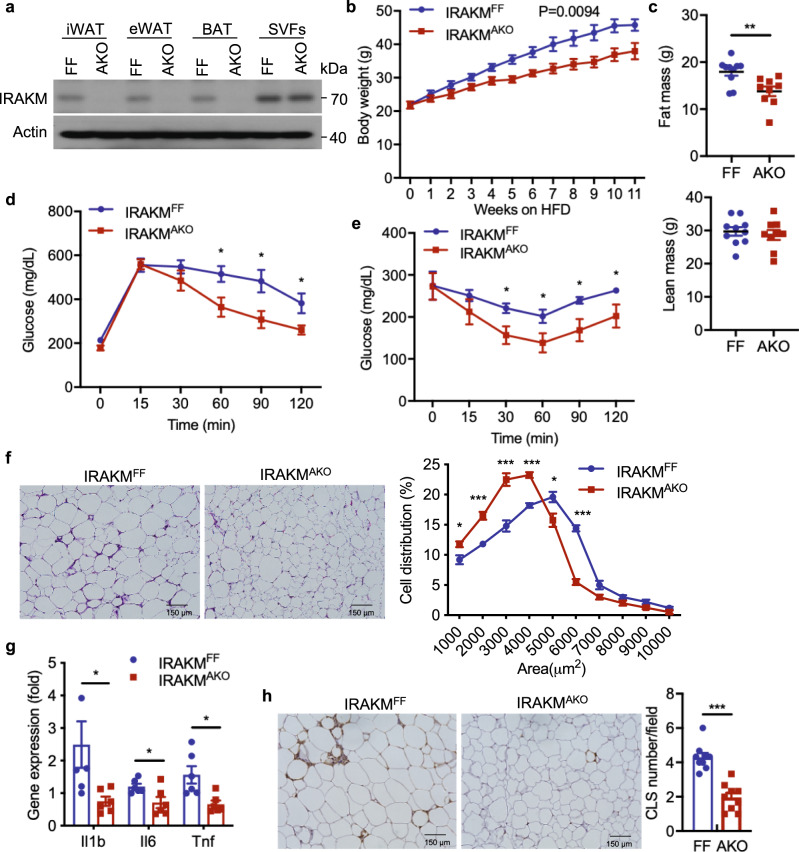
Adipocyte-specific IRAKM deficiency attenuates HFD-induced obesity. **a** Western blot analysis of IRAKM expression in mature adipocytes and stromal vascular fractions (SVFs) of iWAT, eWAT, and BAT from IRAKM^F/F^ and IRAKM^AKO^ mice. Data are representative of two independent experiments. **b** Body weight of IRAKM^F/F^ and IRAKM^AKO^ mice fed with high-fat diet (HFD) for 11 weeks (*n* = 6 males per group). **c** EchoMRI analysis of IRAKM^F/F^ and IRAKM^AKO^ mice after HFD feeding (*n* = 10 for IRAKM^F/F^ mice; *n* = 9 for IRAKM^AKO^ mice; *P* = 0.0058.). **d** Glucose tolerance test (GTT) were analyzed in HFD-fed IRAKM^F/F^ and IRAKM^AKO^ mice (*n*  =  6 mice for each group; from left to right: *P* = 0.023, 0.021, 0.034). **e** Insulin tolerance test (ITT) were analyzed in HFD-fed IRAKM^F/F^ and IRAKM^AKO^ mice (*n* = 7 for IRAKM^F/F^ mice; *n* = 6 for IRAKM^AKO^ mice; from left to right: *P* = 0.017, 0.039, 0.018, 0.037). **f** Representative H&E staining of iWAT sections from HFD-fed IRAKM^F/F^ and IRAKM^AKO^ mice. Adipocyte cell size distributions were analyzed in different sections from five mice (*n* = 5 for IRAKM^F/F^ mice; *n* = 4 for IRAKM^AKO^ mice; 3 sections per mouse; from left to right: *P* = 0.029, 0.00029, 0.0008, 0.00006, 0.027, 0.000006). **g** Inflammatory genes expression in iWAT of HFD-fed IRAKM^F/F^ and IRAKM^AKO^ mice were analyzed by real-time PCR (*n* = 6; from left to right: *P* = 0.038, 0.028, 0.011). **h** Representative Mac2 staining of iWAT sections from HFD-fed mice. Numbers of crown-like structures (CLS) were quantified via Mac2 staining in different sections (*n* = 3 mice per group; 3 views per slide and 3 sections per mouse; *n* = 9 sections; *P* = 0.000009). Statistical significance was determined by two-way ANOVA followed by post hoc analysis (**b**) or two-tailed Student’s *t*-test (**c**–**h**). *P < 0.05. **P < 0.01. All data represent mean ± s.e.m. Source data are provided as the Source data file.

Although IRKAM expression was believed to be restricted in monocytes/macrophages and dendritic cells, we found that IRAKM expression was upregulated during primary adipocyte differentiation from stromal vascular fraction of mice adipose tissues (﻿Supplementary Fig. 1a). To study the adipocyte-intrinsic function of IRAKM in vivo, we generated adipocyte-specific IRAKM-deficient (IRAKM^AKO^) mice by breeding IRAKM^FF^ mice to Adiponectin-Cre transgenic mice. IRAKM protein expression was detected in ﻿epididymal white adipose tissue (eWAT), inguinal white adipose tissue (iWAT), and brown adipose tissue (BAT) of mice (﻿Supplementary Fig. 1b). IRAKM expression was readily detectable in mature adipocytes, although it was significantly less than that in F4/80+ macrophages of iWAT and BAT tissues (﻿Supplementary Fig. 1e). Notably, IRAKM expression was only found in stromal vascular fraction of eWAT, iWAT, and BAT tissues, but not in mature adipocytes from IRAKM^AKO^ mice (Fig. 1a). IRAKM^AKO^ male mice and age-matched male littermate controls were subjected to high-fat diet (HFD, 60% fat) and weight changes were monitored weekly. Compared to control mice, IRAKM^AKO^ mice gained less body weight after HFD feeding (Fig. 1b). EchoMRI analysis showed that adipocyte-specific IRAKM deficiency reduced the fat mass but not lean mass in response to HFD feeding (Fig. 1c), and the weight reduction was observed in eWAT, iWAT, and BAT of HFD-fed IRAKM^AKO^ mice (Supplementary Fig. 1f). In addition, IRAKM deficiency improved glucose tolerance (Fig. 1d) and insulin resistance (Fig. 1e) after HFD feeding. Histological analysis showed that the adipocyte cell sizes were decreased in the iWAT of IRAKM^AKO^ mice compared to that in the wild-type littermate control mice (Fig. 1f). Furthermore, HFD-induced inflammatory gene expression and formation of crown-like structure were reduced in iWAT of IRAKM^AKO^ mice compared to control mice (Fig. 1g, h). However, IRAKM deficiency in adipocytes did not change body weight and insulin resistance upon chow diet feeding (Supplementary Fig. 1g, h). These results indicate that IRAKM has an indispensable role in adipocytes contributing to adipose tissue dysfunction and adiposity.

### Adipocyte-specific IRAKM deficiency improves energy expenditure

**Fig. 2:**
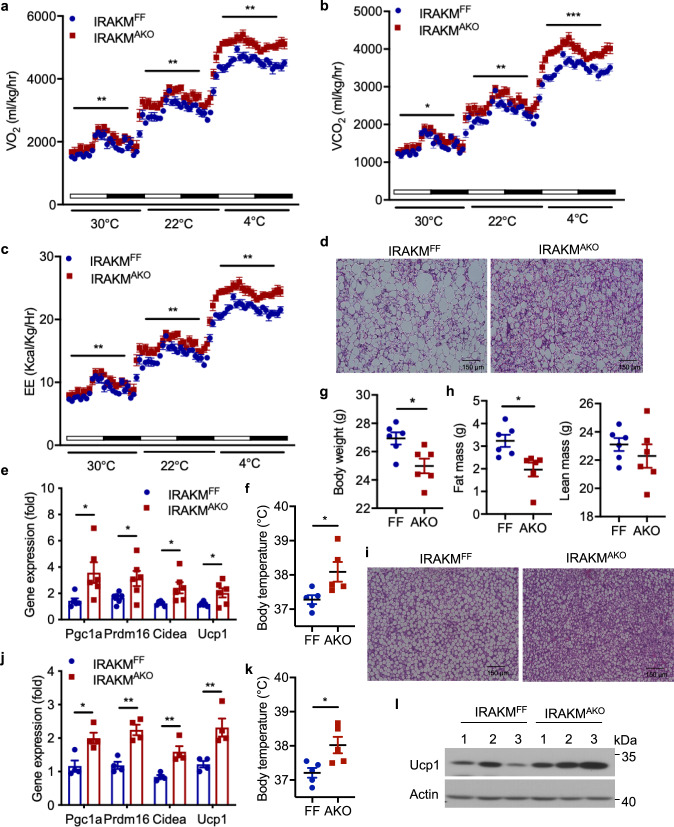
Adipocyte-specific IRAKM deficiency promotes energy expenditure. **a**–**c** VO_2_ (**a**), VCO_2_ levels (**b**), and energy expenditure (EE) (**c**) of HFD-fed IRAKM^F/F^ and IRAKM^AKO^ mice were analyzed by Columbus Oxymax metabolic chambers. 12-h light/dark cycle; 72-h total duration; and each light/dark bar represents 12 h duration (*n* = 5 mice per group; from left to right: **a** *P* = 0.0031, 0.0014, 0.0014; **b** *P* = 0.032, 0.0013, 0.0008; **c** *P* = 0.0044, 0.0019, 0.0012). **d** Representative H&E staining of BAT sections from HFD-fed IRAKM^F/F^ and IRAKM^AKO^ mice (3 views per slide and 3 sections per mouse, *n* = 5 mice per group). **e** Thermogenic genes expression in BAT of HFD-fed IRAKM^F/F^ and IRAKM^AKO^ mice were analyzed by real-time PCR (*n* = 6; from left to right: *P* = 0.024, 0.035, 0.020, 0.038). **f** Rectal temperatures for HFD-fed IRAKM^F/F^ and IRAKM^AKO^ mice (*n* = 5; *P* = 0.033). **g** Body weight of IRAKM^F/F^ and IRAKM^AKO^ mice fed with zero-fat diet (ZFD) for 8 weeks (*n* = 6 males per group; *P* = 0.015). **h** EchoMRI analysis of IRAKM^F/F^ and IRAKM^AKO^ mice after ZFD feeding (*n* = 6; *P* = 0.011). **i** Representative H&E staining of BAT sections from ZFD-fed IRAKM^F/F^ and IRAKM^AKO^ mice (3 views per slide and 3 sections per mouse, *n* = 5 mice per group). **j** Thermogenic genes expression in BAT of ZFD-fed IRAKM^F/F^ and IRAKM^AKO^ mice were analyzed by real-time PCR (*n* = 6; from left to right: *P* = 0.011, 0.001, 0.005, 0.008). **k** Rectal temperatures for ZFD-fed IRAKM^F/F^ and IRAKM^AKO^ mice (*n* = 5; *P* = 0.019). **l** Western blot of Ucp1 in whole BAT tissue lysates from ZFD-fed mice. Data are representative of two independent experiments. Statistical significance was determined by two-way ANOVA (**a**–**c**) or two-tailed Student’s *t*-test (**e**–**h**, **j**, **k**). *P < 0.05. **P < 0.01. ***P < 0.001. All data represent mean ± s.e.m. Source data are provided as the Source data file.

﻿We next investigated the impact of adipocyte-specific IRAKM deficiency on energy expenditure. Interestingly, HFD-fed IRAKM^AKO^ mice exhibited dramatically increased oxygen consumption rate (VO_2_), carbon dioxide production (VCO_2_) and energy expenditure (EE) compared to control mice (Fig. 2a–c). There were comparable food intake and physical activity between HFD-fed IRAKM^AKO^ and control mice (Supplementary Fig. 2a, b). However, chow diet-fed IRAKM^AKO^ and control mice had comparable energy expenditure (Supplementary Fig. 2c). Notably, HFD-fed IRAKM^AKO^ BAT had much less lipid accumulation, accompanied by increased thermogenic gene expression, including Pgc1a, Prdm16, Cidea, and Ucp1, compared to control mice (Fig. 2d, e). Moreover, HFD-fed IRAKM^AKO^ mice displayed higher body temperatures compared with control mice fed the HFD (Fig. 2f). To maximize de novo lipogenesis in vivo, we fed IRAKM^FF^ and IRAKM^AKO^ mice with the high carbohydrate/zero-fat diet (ZFD). Compared to control mice, IRAKM^AKO^ mice gained less body weight after ZFD feeding (Fig. 2g). EchoMRI analysis also indicated that IRAKM^AKO^ mice had much reduced fat mass without affecting lean mass in response to ZFD feeding (Fig. 2h). IRAKM^AKO^ BAT also had much less lipid accumulation compared to that in ZFD-fed control mice (Fig. 2i) with substantially increased thermogenic gene expression, including *Pgc1a*, *Prdm16*, *Cidea*, and *Ucp1* (Fig. 2j). Accordingly, ZFD-fed IRAKM^AKO^ mice displayed higher body temperatures and Ucp1 expression in BAT compared with control mice (Fig. 2k, l). Taken together, these results suggest that adipocyte-derived IRAKM might play an important role in driving HFD-induced adipocyte dysfunction by interfering with de novo lipogenesis and energy expenditure.

### IL-1β induces translocation of IRAKM into mitochondria to promote de novo fatty acid synthesis

**Fig. 3:**
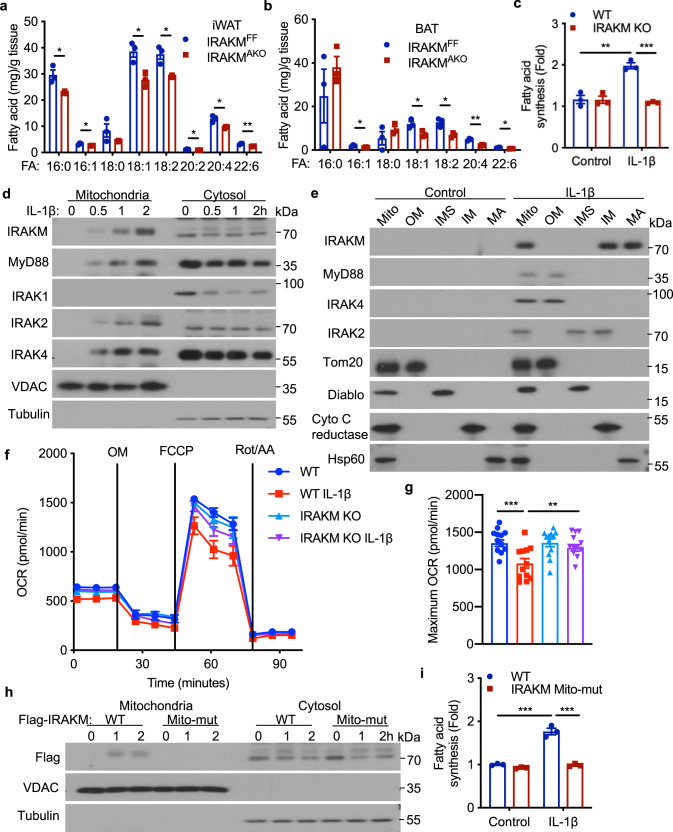
IL-1β induces translocation of IRAKM into mitochondria to promote de novo fatty acid synthesis. **a**, **b** Levels of selected fatty acids (more than 1 mg/g tissues) in iWAT (**a**) and BAT (**b**) from IRAKM^F/F^ and IRAKM^AKO^ mice after HFD feeding were detected with LC-MS/MS analysis (*n* = 3 mice per group; from left to right: **a** *P* = 0.022, 0.025, 0.016, 0.012, 0.03, 0.012, 0.006; **b** *P* = 0.039, 0.032, 0.011, 0.0019, 0.01). **c** De novo fatty acid synthesis measured by the conversion of ^14^C-glucose to lipids in primary adipocytes from WT and IRAKM KO mice treated with or without IL-1β for 24 h (*n* = 3; from left to right: *P* = 0.0026, 0.00025). **d** ﻿Mitochondrial (Mito) and cytosolic (Cyto) fraction were isolated from IL-1β treated primary adipocytes, followed by western blot analysis for indicated proteins. Data are representative of three independent experiments. **e** ﻿Western blot analysis of proteins of mitochondria (Mito), outer mitochondrial membrane (OM), inter mitochondrial membrane space (IMS), inner mitochondrial membrane (IM), and mitochondrial matrix (MA) of mitochondria from IL-1β treated primary adipocytes. Cyto C reductase, Cytochrome C reductase. Data are representative of two independent experiments. **f** OCR in newly differentiated primary WT and IRAKM KO adipocytes treated with or without IL-1β for 24 h (*n* = 4). **g** Maximum OCR in newly differentiated primary WT and IRAKM KO adipocytes treated with or without IL-1β for 24 h (*n* = 12 biologically independent samples; from left to right: *P* = 0.0006, 0.004). **h**, **i** ﻿Flag-tagged wild-type and IRAKM mito-mutant were restored in IRAKM KO primary adipocytes. **h** Mitochondrial (Mito) and cytosolic (Cyto) fraction were isolated from IL-1β treated primary adipocytes, followed by western blot analysis for indicated proteins. Data are representative of three independent experiments. **i** De novo fatty acid synthesis measured by the conversion of ^14^C-glucose to lipids in primary adipocytes treated with or without IL-1β for 24 h (*n* = 3; from left to right: *P* = 0.0005, 0.0005). Statistical significance was determined by two-tailed Student’s *t*-test (**a**–**c**, **g**, **i**). *P < 0.05. **P < 0.01. ***P < 0.001. All data represent mean ± s.e.m. Source data are provided as the Source data file.

Consistent with the fact that HFD-fed IRAKM^AKO^ mice had much less lipid accumulation in BAT and iWAT, the levels of triglycerides and free fatty acids were also substantially reduced in BAT and iWAT of HFD-induced IRAKM^AKO^ mice compared to that of WT mice (Supplementary Fig. 3a, b and Fig. 3a, b). Importantly, IRAKM-deficient white adipocytes (Fig. 3c) and brown adipocytes (Supplementary Fig. 3c) showed reduced de novo fatty acid synthesis in response to IL-1β but not insulin (Supplementary Fig. 3d) stimulation. The critical question was then how IRAKM contributes to IL-1β-induced de novo fatty acid synthesis. We recently reported that IL-1β stimulation induces translocation of MyD88, IRAK4 with IRAK2 into mitochondria in adipocytes^20^. Interestingly, IRAKM was also translocated to mitochondria together with MyD88 and IRAK4 (Fig. 3d). Sub-mitochondrial fractionation analysis showed that IRAKM was localized in the inner membrane and the matrix of mitochondria after IL-1β stimulation, whereas ﻿IRAK2 was translocated into the mitochondrial intermembrane space and localized in inner membrane (Fig. 3e). Furthermore, while cristae formation in mitochondria was not affected by IRAKM deficiency in adipocytes (Supplementary Fig. 3e), IRAKM deficiency abolished IL-1β-induced inhibition of oxidative phosphorylation (Fig. 3f, g). Notably, IRAKM also contains an amphipathic helix structure at its N-terminal, which may function as an IRAKM mitochondria localization signal (Supplementary Fig. 3f). ﻿Mutation of four positive charged amino acids in mitochondria localization signal (MLS) of IRAKM (IRAKM Mito-mut, R56A/K60A/K66A/R70A) greatly diminished IRAKM mitochondria localization (Fig. 3h). TOM20 as the translocator on mitochondrial outer membranes can recognize the hydrophobic surface of the presequence amphipathic helix^28,29^. IRAKM Mito-mut attenuated interaction of IRAKM with TOM20 (Supplementary Fig. 3g). These results suggest that IL-1β stimulation leads to MyD88/IRAK4/IRAKM complex formation and translocation to the outer mitochondrial membrane where IRAKM may then interact with TOM20. The interaction of IRAKM with TOM20 may promote the dissociation of IRAKM from the MyD88-IRAK4 complex, followed by the translocation of IRAKM into mitochondrial inner membranes and matrix. Functionally, IRAKM Mito-mut significantly attenuated IL-1β-induced de novo fatty acid synthesis in primary adipocytes (Fig. 3i). Taken together, these results suggest that IL-1β-induced translocation of IRAKM into mitochondria has a critical impact on IL-1β-induced de novo fatty acid synthesis in adipocytes.

### IRAKM promotes IL-1β-induced mitochondrial citrate transport via interaction with Slc25a1

**Fig. 4:**
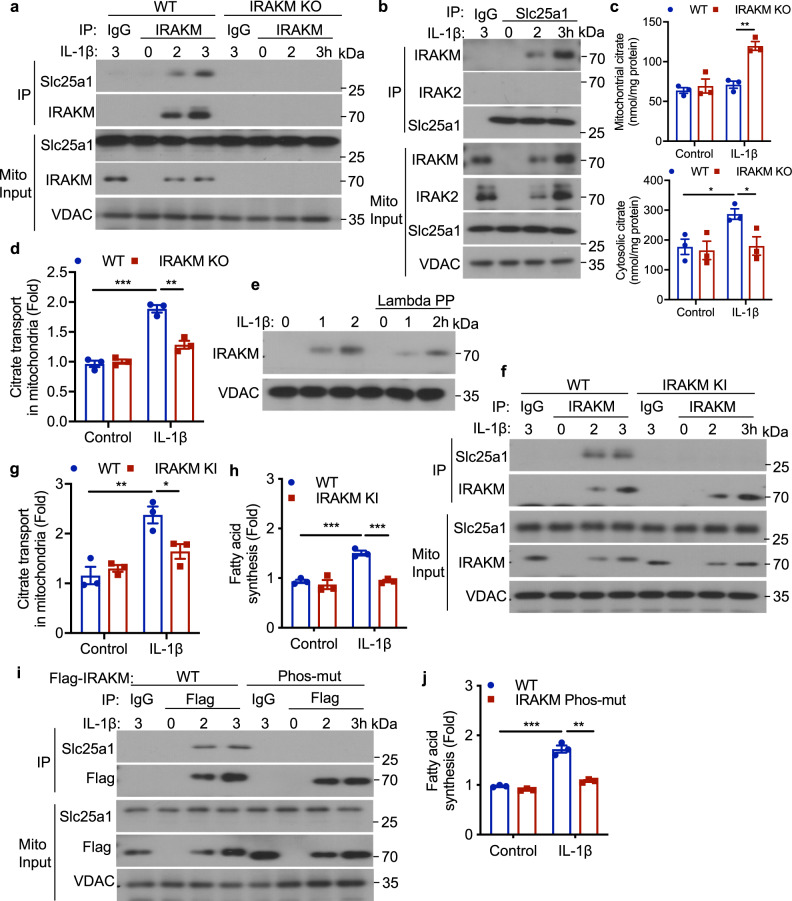
IRAKM promotes IL-1β induced mitochondrial citrate transport via interaction with Slc25a1. **a** Co-immunoprecipitation (IP) analysis was performed with anti-IRAKM antibody in mitochondrial lysate of IL-1β treated primary WT and IRAKM KO adipocytes and followed by western blot analysis for indicated proteins. Data are representative of three independent experiments. **b** Co-immunoprecipitation (IP) analysis was performed with anti-Slc25a1 antibody in mitochondrial lysate of IL-1β treated primary adipocytes and followed by western blot analysis for indicated proteins. Data are representative of three independent experiments. **c** Mitochondrial and cytosolic citrate levels in IL-1β stimulated WT and IRAKM KO primary adipocytes were measured by LC-MS (*n* = 3; mitochondria: *P* = 0.002; cytosol: from left to right: *P* = 0.023, 0.039). **d** Uptake rate of ^14^C-Citrate was measured in intact mitochondria isolated from IL-1β treated primary WT and IRAKM KO adipocytes (*n* = 3; from left to right: *P* = 0.0004, 0.0027). **e** Mitochondria from IL-1β treated primary adipocytes for indicated times was subjected to Lambda phosphatase (Lambda PP) treatment for 30 min, followed by western blot analysis for indicated proteins. Data are representative of three independent experiments. **f** Co-immunoprecipitation (IP) analysis was performed with anti-IRAKM antibody in mitochondrial lysate of IL-1β treated primary WT and IRAKM KI adipocytes and followed by western blot analysis for indicated proteins. Data are representative of three independent experiments. **g** Uptake rate of ^14^C-Citrate was measured in intact mitochondria isolated from IL-1β treated primary WT and IRAKM KI adipocytes (*n* = 3; from left to right: *P* = 0.0075, 0.03). **h** De novo fatty acid synthesis measured by the conversion of ^14^C-glucose to lipids in primary WT and IRAKM KI adipocytes treated with or without IL-1β for 24 h (*n* = 3; from left to right: *P* = 0.0006, 0.0004). **i**, **j** ﻿Flag-tagged wild-type and IRAKM Phos-mut (S167A/S170A) were restored in IRAKM KO primary adipocytes. **i** Co-immunoprecipitation (IP) analysis was performed with anti-Flag antibody in mitochondrial lysate of IL-1β treated primary adipocytes and followed by western blot analysis. Data are representative of three independent experiments. **j** De novo fatty acid synthesis measured by the conversion of ^14^C-glucose to lipids in primary adipocytes treated with or without IL-1β for 24 h (*n* = 3; from left to right: *P* = 0.0005, 0.001). Statistical significance was determined by two-tailed Student’s *t*-test (**c**, **d**, **g**, **h**, **j**). *P < 0.05. **P < 0.01. ***P < 0.001. All data represent mean ± s.e.m. Source data are provided as the Source data file.

We next explored the molecular mechanism for how IRAKM might promote IL-1β-induced de novo fatty acid synthesis in adipocytes. We searched for IRAKM-interacting proteins by proteomic analysis of immunoprecipitants pulled down by anti-IRAKM antibody using cell lysates from untreated and IL-1β-treated adipocytes. Proteomic analysis of IRAKM-interacting proteins revealed that IRAKM interacts with mitochondria citrate transporter Slc25a1 (Supplementary Data). Co-immunoprecipitation experiments validated that IL-1β stimulation was indeed able to induce the interaction of IRAKM (but not IRAK2) with mitochondria citrate transporter Slc25a1 in mitochondrial fraction of primary adipocytes (Fig. 4a, b). Slc25a1 is located within the inner mitochondria membrane, and transports citrate through the impermeable inner mitochondrial membrane in exchange for malate from the cytosol^25^. Once in the cytosol citrate is broken down by ATP-Citrate lyase (ACLY) into acetyl-CoA and oxaloacetate. Since citrate transport from the mitochondria to cytosol is via Slc25a1 and the consequent increase of cytosolic acetyl-CoA is known to drive fatty acid synthesis^30^, we hypothesized that the interaction of IRAKM with Slc25a1 may facilitate citrate transport followed by fatty acid synthesis. Interestingly, we found that IL-1β stimulation indeed induced citrate release from mitochondria to cytosol, which was abolished by IRAKM deficiency (Fig. 4c). ﻿We then measured the transport of radioactive citrate in intact mitochondria isolated from IL-1β-treated WT and IRAKM KO adipocytes, and found that IRAKM deficiency substantially reduced citrate transport in response to IL-1β stimulation (Fig. 4d). Moreover, knockdown of Slc25a1 expression effectively blocked IL-1β-induced fatty acid synthesis (Supplementary Fig. 4a, b).

Interestingly, western blot analysis detected modification of IRAKM in mitochondria fraction, which can be removed by phosphatase treatment, suggesting that IRAKM is probably phosphorylated in mitochondria (Fig. 4e). We have recently found that IRAKM has kinase activity, the modification of IRAKM could be due to auto-phosphorylation^31^. To determine the functional importance of IRAKM kinase activity, we generated IRAKM kinase-inactive mutant (K205A, ATP binding site) knock-in (IRAKM KI) mice. Although IL-1β still induced IRAKM kinase-inactive mutant into mitochondria, ﻿IRAKM kinase inactivation impaired IL-1β-induced IRAKM modification and the interaction of IRAKM with Slc25a1 (Fig. 4f). ﻿IRAKM kinase inactivation attenuated the IRAKM’s impact on citrate transport in mitochondria (Fig. 4g) and IL-1β-induced fatty acid synthesis in white adipocytes (Fig. 4h) and brown adipocytes (Supplementary Fig. 4c). Notably, since IL-1β-induced IRAKM modification correlated with IRAKM’s ability to interact with Slc25a1, it is possible that IRAKM phosphorylation might be required for the interaction of IRAKM with Slc25a1. Our proteomic analysis showed that IRAKM was phosphorylated at Serine 167 and 170 (S167, S170) upon LPS stimulation in macrophages. Mutation of S167/S170 (IRAKM Phos-mut, S167A/S170A) abolished IL-1β-induced IRAKM modification in mitochondria and its interaction with Slc25a1 (Fig. 4i). Moreover, IRAKM Phos-mut substantially reduced IL-1β-induced fatty acid synthesis (Fig. 4j). ﻿These data collectively implicate that IRAKM kinase activity is required for interaction of phosphorylated IRAKM with Slc25a1 in mitochondria to promote fatty acid synthesis.

### IRAKM as a unique kinase phosphorylates Slc25a1 to regulate mitochondrial citrate transport

**Fig. 5:**
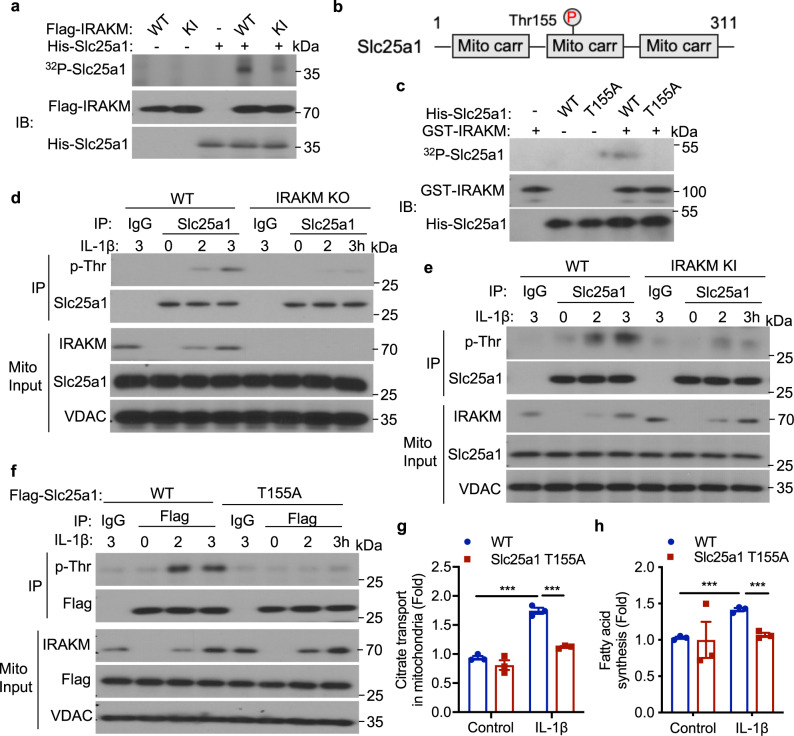
IRAKM as a unique kinase phosphorylates Slc25a1 to regulate mitochondrial citrate transport. **a** In vitro kinase assay of recombinant His-tagged Slc25a1 with Flag-tagged IRAKM WT and kinase-inactive (KI) mutant protein. Data are representative of three independent experiments. **b** Scheme of Slc25a1 protein. Threonine 155 residue was identified to be phosphorylated by IRAKM. **c** In vitro kinase assay of recombinant GST-tagged IRAKM protein with His-tagged Slc25a1 WT and Thr155 non-phosphorylated mutant (T155A). Data are representative of three independent experiments. **d** Co-immunoprecipitation (IP) analysis was performed with anti-Slc25a1 antibody in mitochondrial lysate of IL-1β treated primary WT and IRAKM KO adipocytes and followed by western blot analysis for indicated proteins. Data are representative of three independent experiments. **e** Co-IP analysis was performed with anti-Slc25a1 antibody in mitochondrial lysate of IL-1β treated primary WT and IRAKM KI adipocytes and followed by western blot analysis for indicated proteins. Data are representative of three independent experiments. **f**–**h** Flag-tagged wild-type and Slc25a1 T155A mutant were restored in Slc25a1 KD primary adipocytes. **f** Co-IP analysis was performed with anti-Flag antibody in mitochondrial lysate of IL-1β treated primary adipocytes and followed by western blot analysis for indicated proteins. Data are representative of three independent experiments. **g** Uptake rate of ^14^C-Citrate was measured in intact mitochondria isolated from IL-1β treated primary adipocytes (*n* = 3; from left to right: *P* = 0.0002, 0.0003). **h** De novo fatty acid synthesis measured by the conversion of ^14^C-glucose to lipids in primary adipocytes treated with or without IL-1β for 24 h (*n* = 3; from left to right: *P* = 0.0002, 0.0007). Statistical significance was determined by two-tailed Student’s *t*-test (**g**, **h**). ***P < 0.001. All data represent mean ± s.e.m. Source data are provided as the Source data file.

We next investigated the impact of IRAKM kinase activity on Slc25a1. We found that recombinant IRAKM and Slc25a1 protein interacted with each other (Supplementary Fig. 5a). Importantly, in vitro kinase assay showed that recombinant IRAKM was able to phosphorylate Slc25a1, which was much reduced when the ATP binding site of IRAKM was mutated (Fig. 5a). These results suggest the potential role of IRAKM in phosphorylating Slc25a1. Mass spectrometry analysis revealed that Slc25a1 was phosphorylated at Threonine 155 (Thr155) by recombinant IRAKM in vitro (Supplementary Fig. 5b, Fig. 5b). We then generated Slc25a1 Thr155 non-phosphorylated mutant (T155A), and in vitro kinase assay confirmed that IRAKM phosphorylated Slc25a1 WT but not T155A mutant (Fig. 5c). IL-1β but not insulin (Supplementary Fig. 5c) stimulation indeed induced Slc25a1 phosphorylation in mitochondria of adipocytes, whereas IRAKM deficiency abolished IL-1β-induced Slc25a1 phosphorylation in white adipocytes (Fig. 5d) and brown adipocytes (Supplementary Fig. 5d). Consistently, IRAKM kinase inactivation greatly attenuated IL-1β-induced Slc25a1 phosphorylation in mitochondria of adipocytes (Fig. 5e). IL-1β-induced Slc25a1 phosphorylation was abolished in mitochondria of Slc25a1 knockdown adipocytes restored with Slc25a1 T155A mutant but not WT (Fig. 5f). Moreover, Slc25a1 T155A mutant had much reduced citrate transport activity in response to IL-1β stimulation (Fig. 5g). Likewise, Slc25a1 T155A mutant had substantially reduced IL-1β-induced fatty acid synthesis (Fig. 5h). ﻿Taken together, these data suggest that IL-1β induces mitochondrial IRAKM to interact with and phosphorylate Slc25a1 thereby promoting citrate release from mitochondria to the cytosol for de novo lipid biosynthesis.

### IRAKM is involved in IL-1β induced Pgc1a ﻿acetylation to regulate Ucp1 expression

**Fig. 6:**
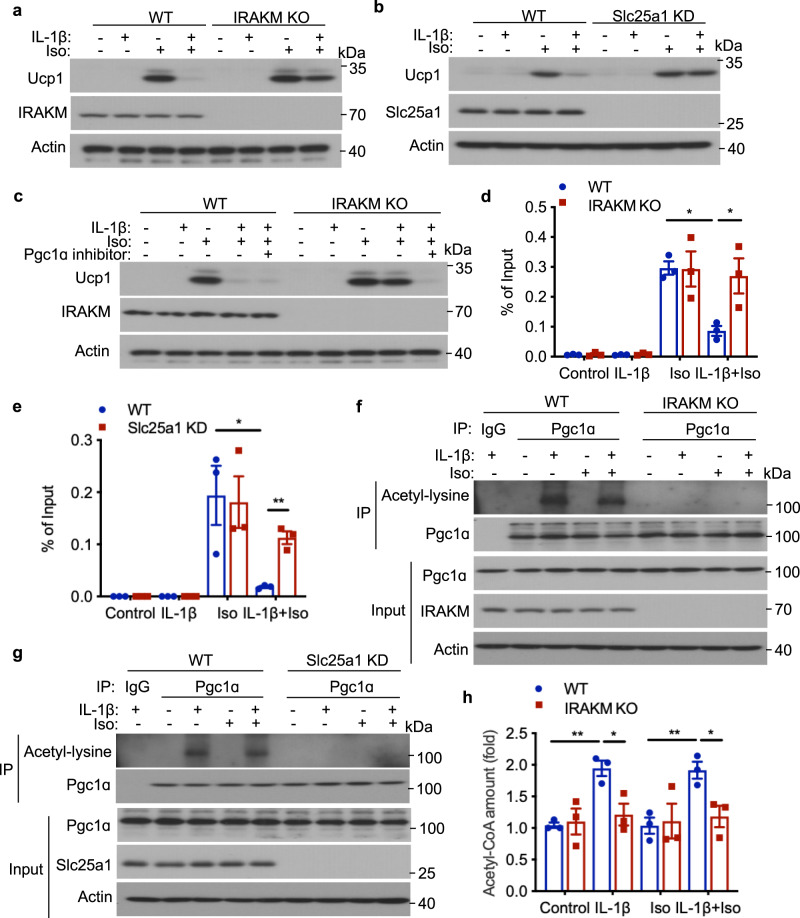
IRAKM is required for IL-1β induced Pgc1a ﻿acetylation to regulate Ucp1 expression. **a** Primary WT and IRAKM KO adipocytes were pre-treated with IL-1β (10 ng/mL) for 2 h, and then stimulated with Isoproterenol (Iso, 10 μM) for 24 h. Western blot analysis was performed for indicated proteins. Data are representative of three independent experiments. **b** Primary WT and Slc25a1 knockdown (KD) adipocytes were pre-treated with IL-1β for 2 h, and then stimulated with Isoproterenol (Iso) for 24 h. Western blot analysis was performed for indicated proteins. Data are representative of three independent experiments. **c** Primary WT and IRAKM KO adipocytes were pre-treated with IL-1β for 2 h, and then stimulated with Isoproterenol (Iso) for 24 h in presence of Pgc1ɑ inhibitor SR-18292 (10 μM). Western blot analysis was performed for indicated proteins. Data are representative of three independent experiments. **d** Chromatin immunoprecipitation (ChIP) was performed with IL-1β pre-treated primary WT and IRAKM KO adipocytes followed by Isoproterenol stimulation for 24 h by Pgc1a antibody. Pgc1a-ChIPed DNAs were examined by qPCR for promoters of Ucp1 (*n* = 3; from left to right: *P* = 0.012, 0.039). **e** ChIP was performed with IL-1β pre-treated primary WT and Slc25a1 KD adipocytes followed by Isoproterenol stimulation for 24 h by Pgc1a antibody. Pgc1a-ChIPed DNAs were examined by qPCR for promoters of Ucp1 (*n* = 3; from left to right: *P* = 0.036, 0.0016). **f** Co-immunoprecipitation (IP) was performed with anti-Pgc1a antibody in cell lysate of IL-1β pre-treated primary WT and IRAKM KO adipocytes followed by Isoproterenol stimulation for 24 h and analyzed by western blot for indicated proteins. Data are representative of three independent experiments. **g** Co-immunoprecipitation (IP) was performed with anti-Pgc1a antibody in cell lysate of IL-1β pre-treated primary WT and Slc25a1 KD adipocytes followed by Isoproterenol stimulation for 24 h and analyzed by western blot for indicated proteins. Data are representative of three independent experiments. **h** Acetyl-CoA levels in IL-1β pre-treated primary WT and IRAKM KO adipocytes followed by Isoproterenol stimulation for 24 h (*n* = 3; from left to right: *P* = 0.0023, 0.025, 0.0089, 0.027). Statistical significance was determined by two-tailed Student’s *t*-test (**d**, **e**, **h**). *P < 0.05. **P < 0.01. All data represent mean ± s.e.m. Source data are provided as the Source data file.

Emerging evidence indicates that de novo lipogenesis might have an impact on the regulation of thermogenic program in adipose tissues^32,33^. Since adipocyte-specific IRAKM deficiency resulted in reduction in de novo lipogenesis with simultaneous induction of thermogenic gene expression including *Ucp1*, we explored the potential underlying mechanism for how IRAKM might link de novo lipogenesis to suppression of thermogenesis in adipocytes. Importantly, while IL-1β had been implicated in modulating Ucp1 expression induced by β-adrenoreceptor agonist (Isoproterenol) in adipocytes^34^, we found that IL-1β-mediated suppression of Isoproterenol-induced Ucp1 expression was abolished by IRAKM deficiency in adipocytes (Fig. 6a). Furthermore, Slc25a1 knockdown also abolished IL-1β-mediated suppression of Isoproterenol-induced Ucp1 expression (Fig. 6b). We then explored the potential cross-talk of IRAKM-Slc25a1 axis with the positive regulators for thermogenic gene program including peroxisome proliferator-activated receptor c coactivator 1a (Pgc1a)^35^. Interestingly, we found that Pgc1a inhibitor abolished Isoproterenol-induced Ucp1 expression that was restored by IRAKM deficiency in IL-1β-treated adipocytes (Fig. 6c). Importantly, the chromatin immunoprecipitation (ChIP) assay indicated that while IL-1β reduced Isoproterenol-induced Pgc1a occupancy on *Ucp1* gene promoter in adipocytes, IRAKM deficiency markedly increased Pgc1a occupancy on *Ucp1* gene (Fig. 6d). Moreover, Slc25a1 knockdown also induced Pgc1a occupancy on *Ucp1* gene in adipocytes (Fig. 6e). These results suggest that IRAKM-Slc25a1 axis has a critical impact on the function of Pgc1a. Pgc1a acetylation has been proposed to suppress Pgc1a transcriptional activity^36–38^. We found that IL-1β induced Pgc1a acetylation in adipocytes, which was completely abolished by IRAKM deficiency (Fig. 6f). Consistently, Slc25a1 knockdown also attenuated IL-1β-induced Pgc1a acetylation in adipocytes (Fig. 6g). Interestingly, while acetyl-CoA is known to mediate Pgc1a acetylation, IL-1β stimulation increased acetyl-CoA levels in adipocytes, which was substantially decreased in adipocytes by IRAKM and Slc25a1 deficiency (Fig. 6h, Supplementary Fig. 6a). ATP citrate lyase (ACLY) is an important enzyme that catalyzes the conversion of citrate to acetyl-CoA in the cytosol of cells. Our results showed that ACLY inhibitor BMS-303141 significantly suppressed IL-1β-induced acetyl-CoA levels and Pgc1a acetylation in adipocytes (Supplementary Fig. 6b, c). Furthermore, BMS-303141 also abolished IL-1β-mediated suppression of Isoproterenol-induced Ucp1 expression in adipocytes (Supplementary Fig. 6d). Moreover, HFD-fed IRAKM^AKO^ iWAT and BAT had much less acetyl-CoA levels, accompanied by decreased Pgc1a acetylation, compared to that in control mice (Supplementary Fig. 6e–g). These results suggest that increased acetyl-CoA level was sufficient to increase Pgc1a acetylation to regulate thermogenic gene expression in adipocytes and in mouse WAT and BAT. These results also implicate that IRAKM may regulate IL-1β-induced acetyl-CoA synthesis via Slc25a1-mediated citrate transport. Taken together, while the IL-1β-IRAKM-Slc25a1 axis promotes de novo lipogenesis via induction of citrate transport followed by fatty acid synthesis, IRAKM-Slc25a1 simultaneously mediates suppression of thermogenic gene expression by elevating acetyl-CoA synthesis and Pgc1a acetylation.

### IRAKM kinase-dead KI mice are protected against obesity

**Fig. 7:**
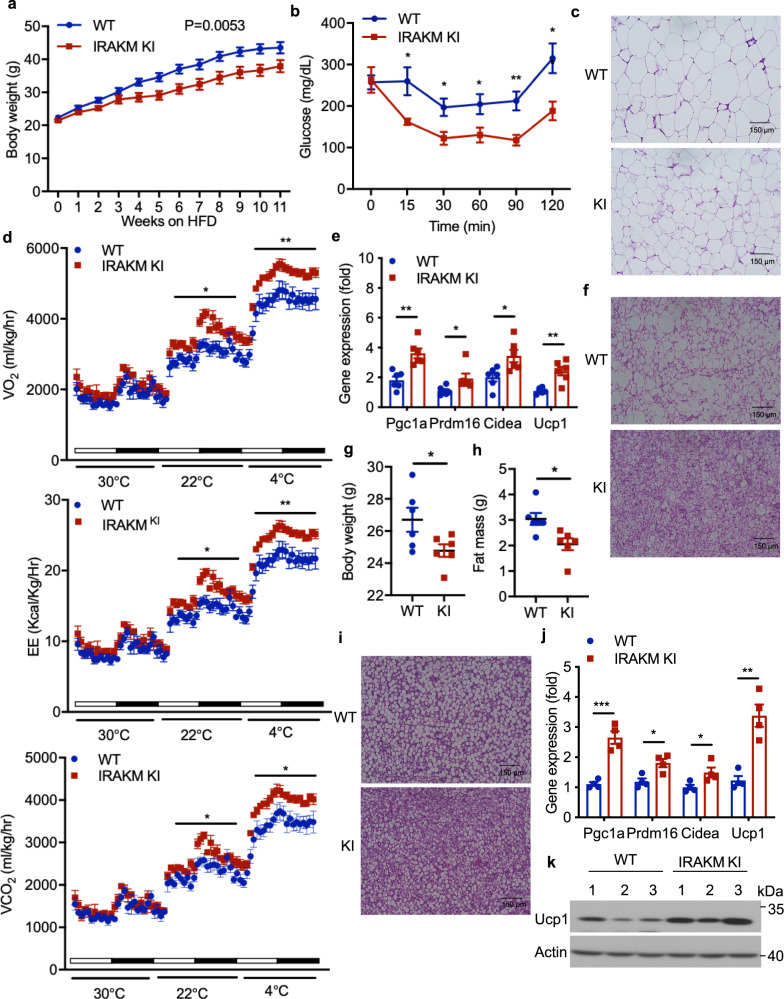
IRAKM kinase-dead KI mice are protected against obesity. **a** Body weight of wide-type (WT) and IRAKM KI mice fed with high-fat diet (HFD) for 11 weeks (*n* = 7 males per group). **b** Insulin tolerance test (ITT) were analyzed in HFD-fed WT and IRAKM KI mice (*n*  =  7 mice for each group; from left to right: *P* = 0.015, 0.013, 0.028, 0.0034, 0.011). **c** Representative H&E staining of iWAT sections from HFD-fed WT and IRAKM KI mice (3 views per slide and 3 sections per mouse, *n* = 5 mice per group). **d** VO_2_, VCO_2_ levels, and energy expenditure (EE) of HFD-fed WT and IRAKM KI mice were analyzed by Columbus Oxymax metabolic chambers. 12-h light/dark cycle; 72-h total duration; and each light/dark bar represents 12 h duration (*n* = 5 mice per group; from left to right: VO_2_: *P* = 0.012, 0.0075; EE: *P* = 0.016, 0.0085; VCO_2_: *P* = 0.029, 0.014). **e** Thermogenic genes expression in BAT of HFD-fed WT and IRAKM KI mice were analyzed by real-time PCR (*n* = 6; from left to right: *P* = 0.0012, 0.039, 0.017, 0.0012). **f** Representative H&E staining of BAT sections from HFD-fed WT and IRAKM KI mice (3 views per slide and 3 sections per mouse, *n* = 5 mice per group). **g** Body weight of WT and IRAKM KI mice fed with zero-fat diet (ZFD) for 8 weeks (*n* = 6 males per group; *P* = 0.046). **h** EchoMRI analysis of fat mass from WT and IRAKM KI mice after ZFD feeding (*n* = 6 males per group; *P* = 0.012). **i** Representative H&E staining of BAT sections from ZFD-fed WT and IRAKM KI mice (3 views per slide and 3 sections per mouse, *n* = 5 mice per group). **j** Thermogenic genes expression in BAT of ZFD-fed WT and IRAKM KI mice were analyzed by real-time PCR (*n* = 6; from left to right: *P* = 0.0004, 0.011, 0.037, 0.0016). **k** Western blot of Ucp1 in whole BAT tissue lysates from ZFD-fed mice. Data are representative of two independent experiments. Statistical significance was determined by two-way ANOVA (**a**, **d**) or two-tailed Student’s *t*-test (**b**, **e**, **g**, **h**, **j**). *P < 0.05. **P < 0.01. ***P < 0.001. All data represent mean ± s.e.m. Source data are provided as the Source data file.

﻿Our results indicate that IRAKM kinase activity is required for IRAKM’s function in mitochondria where IRAKM interacts with and phosphorylates citrate transporter Slc25a1 drive citrate transport followed by fatty acid synthesis and suppression of thermogenic gene expression. These results implicate the importance of IRAKM kinase activity in IRAKM’s contribution to adipose tissue dysfunction and obesity, which can be further exploited to develop potential therapeutic strategy for treating obesity. Therefore, we then examined the role of IRAKM kinase activity in HFD-induced obesity. Consistent with IRAKM-deficient mice, IRAKM kinase-inactive knock-in mice (IRAKM KI) gained less weight (Fig. 7a) with much less fat mass (Supplementary Fig. 7a) compared to the wild-type control mice under HFD. In addition, IRAKM kinase inactivation improved glucose tolerance (Supplementary Fig. 7b) and insulin resistance (Fig. 7b) after HFD feeding. Adipocyte cell sizes were decreased in iWAT (Fig. 7c), and concentration of triglycerides (Supplementary Fig. 7c) were substantially reduced in BAT and iWAT of IRAKM KI mice compared to that in WT mice. HFD-induced formation of crown-like structure and inflammatory gene expression were reduced in iWAT of IRAKM KI mice compared to control mice (Supplementary Fig. 7d, e). Furthermore, HFD-fed IRAKM KI mice exhibited dramatically increased oxygen consumption rate (OCR), carbon dioxide production (VCO_2_), and energy expenditure (EE) at 22 °C and 4 °C compared to control mice (Fig. 7d). Moreover, HFD-fed IRAKM KI mice had much less lipid accumulation in BAT and increased thermogenic gene expression in iWAT and BAT, including *Pgc1a*, *Prdm16*, *Cidea*, and *Ucp1*, compared to that in control mice (Fig. 7e, f, Supplementary Fig. 7f). HFD-fed IRAKM KI mice also displayed higher body temperatures compared with control mice (Supplementary Fig. 7g). WT and IRAKM KI mice were also fed with the high carbohydrate/zero-fat diet (ZFD) to maximize de novo lipogenesis. Compared to control mice, IRAKM KI mice gained less body weight and much less fat mass without impacting lean mass after ZFD feeding (Fig. 7g, h, Supplementary Fig. 7h). Consistently, IRAKM KI BAT had much reduced lipid accumulation (Fig. 7i). Thermogenic genes expression was increased (including *Pgc1a*, *Prdm16*, *Cidea*, and *Ucp1*) in iWAT (Supplementary Fig. 7i) and BAT (Fig. 7j, k) of ZFD-fed IRAKM KI mice accompanied with higher body temperature (Supplementary Fig. 7j). Consistent with IRAKM^AKO^ mice, HFD-fed IRAKM KI iWAT and BAT had much less acetyl-CoA, accompanied by reduced Pgc1a acetylation, compared to control mice (Supplementary Fig. 7k–m). These results indicate that IRAKM kinase activity contributes to HFD-induced obesity, which provides the proof of concept data for IRAKM to be pursued as a drug target for treating obesity in the future.

## Discussion

We report here that IL-1β-IL-1R signaling reprograms adipocyte metabolism by driving the recruitment of IRAKM Myddosome to mitochondria, promoting transport of mitochondrial citrate to cytosol and de novo fatty acid synthesis. In response to IL-1β stimulation, IRAKM, but not its kinase-inactive mutant, enhanced mitochondrial citrate transport to cytosol and de novo lipogenesis via interaction with mitochondrial citrate transporter Slc25a1 in adipocytes. Moreover, Slc25a1 phosphorylation mediated by IRAKM was required for IL-1β-induced transport of mitochondrial citrate to cytosol and consequent de novo lipogenesis in adipocytes. Interestingly, Pgc1a ﻿acetylation was induced by IL-1β via IRAKM-Slc25a1 axis to regulate thermogenic gene expression in adipocytes. Taken together, our results suggest that IRAKM Myddosome mediates the IL-1R-IRAKM-Slc25a1 signaling axis to directly reprogram adipocyte citrate metabolism, resulting in lipid accumulation and suppression of thermogenic gene program.

In support of the critical role of IRAKM Myddosome in adipocyte metabolism, we found that adipocyte-specific deficiency of IRAKM reduced high-fat diet-induced body weight gain, increased whole-body energy expenditure with improved insulin resistance. Consistently, ablation of IRAKM in adipocytes attenuated adipocyte hypertrophy in both WAT and BAT from HFD-fed mice, which is accompanied by reduced lipid accumulation. Notably, adipocyte-specific IRAKM-deficient mice had increased oxygen consumption and energy expenditure compared to that of control mice after HFD feeding. Furthermore, there were increased expression of thermogenic genes and decreased expression of inflammatory genes in BAT of HFD-fed adipocyte-specific IRAKM-deficient mice compared to that of the control mice. Moreover, adipocyte-specific deficiency of IRAKM reduced high carbohydrate/zero-fat diet (ZFD)-induced adipocyte hypertrophy (especially lipid accumulation) and improved thermogenesis in adipose tissues, which echoes the effect of IRAKM on de novo lipogenesis in adipocytes. Collectively, these results implicate a critical impact of IRAKM on obesity-associated adipocyte dysfunction. However, previous studies showed that ﻿IRAKM in monocytes was a key inhibitor of inflammation in obesity and metabolic syndrome of human subjects, and IRAKM deficiency in ﻿nonobese diabetic mice ﻿enhanced activation and antigen-presenting function of dendritic cells to promote development of type 1 diabetes^39,40^. Therefore, future studies are required to investigate cell type-specific roles of IRAKM (especially monocytes/macrophages) in obesity.

Our published study and results here indicate that IL-1β stimulation induced translocation of MyD88, IRAK4 with IRAK2 and IRAKM, but not IRAK1, to mitochondria. TOM20 is the major receptor responsible for the recognition and translocation of cytosolically synthesized proteins into mitochondria. Nuclear magnetic resonance (NMR) and crystal structures of the TOM20 receptor domain demonstrated that TOM20 recognizes the hydrophobic surface of the presequence amphipathic helix [also known as mitochondria localization signal (MLS)]. Presequences reside in the first 10–90 N-terminal residues, exhibit a high composition of arginine/lysine and near absence of negatively charged residues^28,29^. By using the NetWheels program, we identified such an amphipathic helix structure (MLS) at the N-terminus of IRAK2 and IRAKM, which contains 4 arginine/lysine residues and only one acidic reduce. However, the corresponding amphipathic helix in IRAK1 contains 4 acid residues. These findings suggest that the presence of strong MLS at the N-terminal of IRAK2 and M (but not IRAK1) allow their mitochondrial translocation in response to IL-1β stimulation. IL-1β induced the interaction of IRAKM with TOM20. We previously reported that mutation of the positively charged amino acids in MLS of IRAK2 (IRAK2 Mito-mut) greatly reduced the interaction of IRAK2 with TOM20 and diminished IRAK2 mitochondria localization^20^. Likewise, IRAKM Mito-mut also attenuated interaction of IRAKM with TOM20 and significantly attenuated IL-1β-induced IRAKM mitochondria localization, de novo lipogenesis in primary adipocytes. Sub-mitochondrial fractionation analysis showed that, in contrast to localization of MyD88 and IRAK4 in the outer membrane fraction, IRAKM was localized in the inner membrane and the matrix of mitochondria after IL-1β stimulation, while ﻿IRAK2 was translocated into the mitochondrial intermembrane space and localized in inner membrane (Fig. 3e). These findings suggest that the interaction of IRAK2 and IRAKM with TOM20 may promote their dissociation from the MyD88-IRAK4 complex, followed by their specific translocation into the inner membrane, mitochondrial intermembrane space, or matrix. Notably, consistent with their differential sub-mitochondrial localization, IRAKM and IRAK2 have their independent interacting partners to carry out distinct biochemical activity and cell metabolism. IRAKM (but not IRAK2) interacts with Slc25a1 to promote IL-1β-induced transport of mitochondrial citrate to cytosol and consequent de novo lipogenesis in adipocytes. Our recent publication^20^ and data here indeed indicate the non-redundant roles of IRAK2 and IRAKM in adipocyte metabolism. Notably, IL-1β-induced supercomplex formation and suppression of fatty acid oxidation (FAO) were dependent on IRAK2^20^ but not IRAKM (Supplementary Fig. 4d). On the other hand, IL-1β stimulation induced the interaction of IRAKM (but not IRAK2) with mitochondria citrate transporter Slc25a1 in adipocytes for IL-1β-induced transport of mitochondrial citrate to cytosol and consequent de novo lipogenesis in adipocytes (Fig. 4a, b). Therefore, these data suggested the non-redundant role of IRAKM in Slc25a1-mediated de novo lipogenesis to control lipid metabolism in adipocytes, contributing to obesity. It is important to notice that although IRAK2 does not interact with Slc25a1, IRAK2 deficiency still affected fatty acid synthesis (Supplementary Fig. 4f). Therefore, both IRAK2 and IRAKM have critical roles in lipid metabolism. It will be interesting and important to further investigate their redundant and possible cooperative roles in lipid metabolism.

Chronic ﻿low-grade inflammation contributes to adipose tissue dysfunction, insulin resistance, and metabolic disease^6,7^. ﻿Notably, there is a strong positive correlation between adipocyte size and adipose tissue inflammation in white adipose tissue^41,42^. IL-1β is elevated in adipose tissue from diabetic patients compared to that of non-diabetic people and also induced by HFD in mouse adipose tissue^20^. Our results here implicated that IL-1β was able to induce de novo fatty acid synthesis to promote lipid accumulation in adipocytes. IRAKM was required for IL-1β induced de novo fatty acid synthesis in adipocytes, and IRAKM deficiency in adipocytes reduced lipid accumulation in both WAT and BAT from HFD-fed mice. Mechanistically, IL-1β stimulation induced IRAKM translocation into mitochondria where IRAKM interacts with mitochondrial citrate transporter Slc25a1 in adipocytes. Slc25a1 as a mitochondrial citrate transporter is localized in the inner mitochondrial membrane and promotes the efflux of citrate to the cytoplasm in exchange for cytosolic malate^43–45^. Slc25a1 activity has been linked to several pathologic conditions including cancer, aging, and developmental disorders^46–49^. Recent study reported that Slc25a1 serves as an important player in the pathogenesis of fatty liver disease and ﻿high-fat diet-induced obesity^50^. Interestingly, we found that interaction of IRAKM with Slc25a1 promoted mitochondrial citrate transport to cytosol, resulting in de novo fatty acid synthesis in adipocytes. Interestingly, while acetyl-CoA is known to mediate Pgc1a acetylation, IL-1β stimulation increased acetyl-CoA levels and Pgc1a ﻿acetylation in adipocytes, which were substantially decreased in adipocytes by IRAKM and Slc25a1 deficiency. Furthermore, IL-1β-induced Pgc1a ﻿acetylation reduced the transcriptional activity of Pgc1a in driving thermogenic gene expression in adipocytes. Therefore, while enhancing de novo lipogenesis via induction of citrate transport followed by fatty acid synthesis, IRAKM-Slc25a1 simultaneously suppresses thermogenic gene expression by elevating acetyl-CoA synthesis and Pgc1a acetylation. Taken together, these results provide the mechanistic insight into how de novo lipogenesis can be intrinsically linked to the modulation of thermogenic gene program in adipocytes.

More importantly, IRAKM-mediated Slc25a1 phosphorylation was required for IL-1β-induced transport of mitochondrial citrate to cytosol and de novo fatty acid synthesis in adipocytes. Unexpectedly, though IRAKM kinase-inactive mutant was still translocated into the mitochondria, kinase-inactive IRAKM lost the ability to interact with and phosphorylate Slc25a1, which was accompanied by the loss of IL-1β-induced IRAKM modification. Furthermore, kinase-inactive IRAKM reduced IL-1β-induced de novo fatty acid synthesis in adipocytes, and lipid accumulation in both WAT and BAT from HFD-fed mice to improve obesity-related pathophysiology. Our results indicated that IRAKM as a unique kinase for mitochondrial citrate transporter Slc25a1 links mitochondrial metabolism with fatty acid synthesis in adipocytes. The findings here suggest that specifically disrupt interactions of IRAKM with Slc25a1 or specific IRAKM inhibitors might offer potential therapeutic strategies for the treatment of obesity-associated pathologies.

## Methods

### Antibodies

Antibodies used for immunoblotting, and immunohistochemistry were as follows: rabbit polyclonal anti-Slc25a1 (1 μg for IP, 1:1000 for WB) (15235-1-AP) and mouse monoclonal Slc25a1 (1:1000) (66771-1-Ig) were purchased from Proteintech. Goat polyclonal anti-IRAKM (1 μg for IP, 1:1000 for WB) (PAB7483) was obtained from Abnova. Rabbit polyclonal anti-Pgc1α (1 μg for IP, 1:1000 for WB) (AB3242) was purchased from Millipore. Mouse monoclonal anti-Ucp1 (1:1000) (MAB6158) was obtained from R&D Systems. Rabbit polyclonal anti-IRAK2 (1:1000) (ab62419), Mouse monoclonal anti-Cytochrome C reductase (1:1000) (ab110252), and Mouse monoclonal anti-Mac2 (1:50 for IHC) (ab2785) were purchased from Abcam. Mouse monoclonal anti-Actin (1:1000) (sc-8432), Mouse monoclonal anti-VDAC (1:1000) (sc-390996), Mouse monoclonal anti-Tubulin (1:1000) (sc-166729), and Mouse monoclonal anti-TOM20 (1:1000) (sc-17764) were obtained from Santa Cruz Biotechnology. Rabbit monoclonal anti-IRAK1 (1:1000) (4504), Rabbit polyclonal anti-IRAK4 (1:1000) (4363), Rabbit monoclonal anti-MyD88 (1:1000) (4283), Mouse monoclonal anti-Diablo (1:1000) (2954), Rabbit polyclonal anti-Hsp60 (1:1000) (4870), Rabbit monoclonal anti-Flag (1:1000) (14793), Rabbit monoclonal anti-His (1:1000) (12698), Rabbit monoclonal anti-GST (1:1000) (2625), Mouse monoclonal anti-Phospho-Threonine (1:1000) (9386), and Rabbit polyclonal anti-Acetylated-Lysine (1:1000) (9441) were purchased from Cell Signaling Technology.

### Mouse models

IRAKM knockout mice were generated by Dr. Richard Flavell (Yale School of Medicine, New Haven) as described^51^. IRAKM flox/flox mice were generated by our lab as described^52^. ﻿Adipocyte-specific deletion of IRAKM (IRAKM^AKO^) was obtained by breeding IRAKM flox/flox mice with Adiponectin-Cre transgenic mice (Jackson Laboratory, 028020). IRAKM kinase-dead(K205A) knock-in mice were generated by CRISPR/Cas-mediated genome engineering (Cyagen Biosciences). Six- to eight-week-old male mice were maintained on a high-fat diet composed of 60% kcal derived from fat (TD06414, Envigo) for 12 weeks, or on high carbohydrate (62% Sucrose)/zero-fat diet (TD03314, Envigo) for 8 weeks while normal mice maintained on either standard rodent chow diet (2918 Teklad Global 18% Protein Rodent Diet, Envigo). Animals were housed in a specific pathogen-free facility at a temperature of 21 °C, relative humidity of 50–70%, and under a constant 12-h light/dark cycle and given free access to food and water. All procedures using animals were approved by the Institutional Animal Care and Use Committee (IACUC) of Cleveland Clinic (protocol number: 2020–2316). Ethical compliance with IACUC protocols and institute standards was maintained.

### ﻿Primary adipocyte culture

Subcutaneous and brown adipose tissues from 6- to 8-week-old mice were minced into small pieces, followed by incubation with ﻿1.5 u/ml Collagenase D (Roche), and 2.4 u/ml ﻿Dispase II in PBS buffer for 40–50 min in a 37 °C shaker water bath. ﻿ Digestion was stopped by adding 5 ml DMEM/F12 complete medium (DMEM/F12 containing 10% FBS and Penicillin/Streptomycin), and cell suspension buffer was filtered through 100 μm cell strainer and centrifugated for 10 min at 700 × *g* to get the stromal vascular fraction pellet. For white adipocytes differentiation, stromal vascular fraction was cultured in DMEM/F12 complete medium to 90–95% confluence, and then differentiated in DMEM-F12 complete medium containing (1 μM dexamethasone, 0.5 mM IBMX, 5 μg/ml insulin, and ﻿0.5 μM rosiglitazone) (Sigma-Aldrich) for 2 days. Cells were then changed for DMEM-F12 complete medium containing 5 μg/ml insulin every 2–3 days until ﻿more than 90% cells were fully differentiated to mature adipocytes﻿ before the following experiment. For brown adipocytes differentiation, stromal vascular fraction was cultured in DMEM/F12 complete medium to 90–95% confluence, and then differentiated in DMEM-F12 complete medium containing (125 μM Indomethacin, 1 μM dexamethasone, 0.5 mM IBMX, 5 μg/ml insulin, 1 nM T3, and ﻿0.5 μM rosiglitazone) for 2 days. Cells were then changed for DMEM-F12 complete medium containing 5 μg/ml insulin, 1 nM T3, and ﻿0.5 μM rosiglitazone every 2–3 days until ﻿more than 90% cells were fully differentiated to mature adipocytes﻿ before the following experiment.

### Mature adipocytes and F4/80+ macrophages isolation

Adipose tissues were enzymatically digested as above. After centrifugation, mature adipocyte layer was on the top, and stromal vascular fraction (SVF) pellet was on the bottom of the tube. SVF was then purified with anti-F4/80 MicroBeads (Miltenyi Biotec) for macrophages. Mature adipocytes and F4/80+ macrophages were eventually lysed in TRIzol (Invitrogen) for further analysis.

### Mitochondria isolation

Mitochondrial isolation was performed according to the following protocol^53^. Briefly, primary adipocytes were homogenized in 2 ml ice-cold isolation buffer (10 μM Tris–MOPS, 10 μM EGTA, and 0.2 M sucrose, pH 7.4) using a Teflon Potter-Elvehjem homogenizer. After homogenization, the specimen was centrifugated at 500 × *g* for 10 min, the mitochondrial fractions were further washed and pelleted at 7000 × *g* for 10 min for 3 times. The mitochondrial pellets were lysed in NP-40 lysis buffer (﻿1% NP-40, 20 mM Tris-HCl pH 7.4, 150 mM NaCl, and EDTA-free protease inhibitors) following co-immunoprecipitation and western blot analysis.

### Mitochondrial sub-fractionation

﻿Separation of inner and outer mitochondrial membranes was performed following the protocol below^54–56^. Mitochondria of primary adipocytes from 30 15-cm cell culture plates were resuspended in hypotonic medium (10 mM KCl, 2 mM HEPES, pH 7.2) with gentle stirring for 20 min on ice. One-third volume of hypertonic medium (1.8 mM sucrose, 2 mM ATP, 2 mM MgSO_4_, 2 mM HEPES, pH 7.2) was then added and the solution stirred for an additional 5 min. The mitochondria were sonicated for 15 s at 3 amps before being layered on top of a stepwise gradient of 0.76, 1.0, 1.32, and 1.8 M sucrose and spun at 75,000 × *g* for 3 h in an SW40 Ti rotor (Beckman). The soluble intermembrane space (IMS) fraction was collected from the upper supernatant, the outer membrane (OM) between the 0.76 and 1.0 M interface, and the mitoplasts (MP) from the pellet. The MP and OM fractions were washed with MSHE and re-pelleted (MP at 10,000 × *g* for 10 min, OM at 120,000 × *g* for 45 min). MP was sonicated 3 × 2 min on ice with 1-min intervals. The solution was spun at 15,000 × *g* to remove intact MP, and the resulting supernatant was spun at 120,000 × *g* for 45 min at 4 °C to pellet inner membrane (IM). The soluble matrix (SM) fraction was collected from the supernatant. Different fractionations were eventually eluted and boiled with 4xLaemmli sample buffer for western blot analysis.

### Immunochemistry

Tissues were dissected, and then fixed in 10% formalin. Paraffin-embedding tissues were sectioned and subject to H&E staining at Lerner Research Institute histology core. For Mac2 staining, paraffin-embedded formalin-fixed iWAT depots were sectioned at 5 μm. After antigen retrieval with sodium citrate buffer (pH 6.0) and blocking with 3% BSA in PBS solution, mouse anti-Mac2 monoclonal antibody (dilution: 1:50) was incubated at 4 °C overnight. The antibody was visualized using rat anti-mouse HRP-conjugated secondary antibody and the DAB substrate detection kit (BD Pharmingen). The slides were counterstained with hematoxylin and mounted, then imaged with a light microscope (Keyence). Size of adipocytes was determined by ImageJ (version 1.53i 24) analysis of H&E stained tissues.

### Co-immunoprecipitation

Cells were harvested and lysed on ice in a lysis buffer containing 0.5% NP-40, 20 mM Tris-HCl pH 7.4, 150 mM NaCl, and EDTA-free protease inhibitors for 20 min, followed by centrifugation at 12,000 × *g* for 15 min to extract clear lysates. For immunoprecipitation, cell lysates were incubated with 1 μg indicated antibodies and protein G-sepharose beads at 4 °C overnight. After incubation, the beads were washed four times with lysis buffer and the precipitates were eluted with 2xLaemmli sample buffer. Elutes and whole-cell extracts were resolved on SDS-PAGE followed by western blot with the indicated antibodies.

### Lambda protein phosphatase treatment

Isolated mitochondria were lysed in NP-40 lysis buffer (﻿1% NP-40, 20 mM Tris-HCl pH 7.4, 150 mM NaCl, and EDTA-free protease inhibitors) and incubated with lambda protein phosphatase (NEB) at 30 °C for 30 min. After incubation, mitochondrial lysates were eventually boiled with 4xLaemmli sample buffer for western blot analysis.

### Site-direct mutagenesis

All pMX-Flag-IRAKM WT, IRAKM (R56A/K60A/K66A/R70A), IRAKM (K205A), and IRAKM (S167A/S170A), pMX-Flag-Slc215a1 WT, and Slc215a1 (T155A), pET28-His-Flag-Slc215a1 WT, and Slc215a1 (T155A) plasmids were generated using Q5^®^ Site-Directed Mutagenesis Kit (NEB) according to the manufacturer’s instruction. Mutations were confirmed by DNA sequencing.

### Protein purification

3×Flag-IRAKM WT and kinase-dead mutant protein were purified from HEK293T cells transfected with 3×Flag-IRAKM WT and kinase-dead mutant IRAKM (K205A). Cells were lysed in NP-40 lysis buffer. The cell lysates were incubated with anti-Flag beads at 4 °C. Beads were washed four times with NP-40 lysis buffer and eluted with 0.5 mg/ml 3×Flag peptide in TBS.

For His and sumo-tagged Slc25a1 WT and non-phosphorylated mutant (T155A) protein purification, *E. coli* BL21 cells were transformed with pET28a-Slc25a1 WT and T155A plasmid. The bacterial culture was grown until it reached at the OD600 value of 0.6–0.8. 0.1 mM of IPTG was added for 4–5 h to induce protein expression at 37 °C. Bacterial cells were harvested, resuspended, and lysed in BugBuster master mix buffer (Millipore). His-tagged proteins were purified with Ni-NTA column (GE Healthcare) according to the manufacturer’s instruction.

### In vitro kinase assay

Recombinant IRAKM and Slc25a1 proteins were incubated with 30 μl kinase buffer including 100 μM of cold ATP, 5 μCi of radioactive ATP (γ-^32^P ATP) at 30 °C for 60 min. The reaction was stopped by the addition of 4x sample buffer and heating for 5 min at 95 °C. Proteins were subjected to SDS-PAGE, followed by autoradiography.

### Identification of Slc25a1 phosphorylation induced by IRAKM

Recombinant IRAKM and Slc25a1 proteins were incubated for in vitro kinase assay, and fractionated on an SDS-Page gel. In-gel digestion followed by LC-MS/MS was performed by the Proteomics Core at Lerner Research Institute. A small area around the bait protein was cut into three bands. For the protein digestion, these bands were divided into a number of smaller pieces. The gel pieces were washed with water and dehydrated in acetonitrile. The bands were then reduced with DTT and alkylated with iodoacetamide prior to the in-gel digestion. All bands were digested in-gel by adding 5 μL 10 ng/μL trypsin or chymotrypsin in 50 mM ammonium bicarbonate and incubating overnight at room temperature. The peptides that were formed were extracted from the polyacrylamide in two aliquots of 30 μL 50% acetonitrile with 5% formic acid. The digests from the same sample were combined and dried down in speedvac, reconstituted in 50 μl binding buffer (1 M glycolic acid in 80% acetonitrile, 5% trifluoroacetic acid), and proceeded with phosphopeptides enrichment using TiO2 Mag Sepharose®. After enrichment, the final eluate was dried down in speedvac and reconstituted in 15 μl of 1% acetic acid for LC-MS/MS. Digested peptides were analyzed on a ThermoFisher Scientific UltiMate 3000 HPLC system (ThermoFisher Scientific, Bremen, Germany) interfaced with a ThermoFisher Scientific Orbitrap Fusion Lumos Tribrid mass spectrometer (Thermo Scientific, Bremen, Germany). Liquid chromatography was performed prior to MS/MS analysis for peptide separation. The HPLC column used is a Dionex 15 cm × 75 µm Acclaim Pepmap C18, 2 μm, 100 Å reversed-phase capillary chromatography column. 5 μL volumes of the peptide extract were injected and peptides eluted from the column by a 1100-min acetonitrile/0.1% formic acid gradient at a flow rate of 0.30 μL/min and introduced to the source of the mass spectrometer online. Nano electrospray ion source was operated at 2.3 kV. The digest was analyzed using the data-dependent multitask capability of the instrument acquiring full scan mass spectra using a Fourier Transform (FT) orbitrap analyzer to determine peptide molecular weights and collision-induced dissociation (CID) MS/MS product ion spectra with an ion-trap analyzer to determine the amino acid sequence in successive instrument scans. The MS method used in this study was a data-dependent acquisition (DIA) with 3 s duty cycle. It includes one full scan at a resolution of 120,000 followed by as many MS/MS scans as possible on the most abundant ions in that full scan. Dynamic exclusion was enabled with a repeat count of 1 and ions within 10 ppm of the fragmented mass were excluded for 60 s. For the identification of Slc25a1 phosphorylation sites, the tryptic and chymotryptic data was analyzed using Sequest bundled into Proteome Discoverer V2.3. The LC-MS/MS data were searched specifically against the sequence of Slc25a1 with the same parameters as above except phosphorylation was considered as a variable modification and for the chymotryptic digests, semi-chymotryptic peptides with a maximum of 4 missed cleavages were considered. For modification site identification, all sites were identified using the ptmRS node in PD2.3 and all identified sites were subjected to manual validation.

### Proteomic analysis of IRAKM-interacting proteins

Primary adipocytes were stimulated with IL-1β for 1 h, and then immunoprecipitated using an anti-IRAKM antibody for proteomic analysis. Samples included untreated, and IL-1β-treated adipocytes groups (*n* = 1). Samples were submitted for in-solution digestion in the Proteomics Core at Lerner Research Institute. The sample was filtered using a 3 K Amicon Ultra 0.5 mL centrifugal filter, dried in a speedvac and reconstituted in 50 μL of 6 M urea buffer. The protein sample was reduced with DTT and alkylated with iodoacetamide. The sample was digested by adding Trypsin to the sample at a 25:1 protein:protease ratio (w/w). The sample was mixed and incubate for 3–4 h at 37 °C. The sample was then diluted 6-fold with 50 mM Tris-HCl (pH 8) to reduce urea concentration to 1 M or below. Digestion was continued overnight at 37 °C. The digestion was terminated by adding TFA to a final concentration of 0.5–1%. The sample was desalted using a PepClean C18 spin column and resuspended in 1% acetic acid to make up a final volume of ~30 μL for LC-MS analysis. Digested peptides were analyzed on a ThermoFisher Scientific UltiMate 3000 HPLC system (ThermoFisher Scientific, Bremen, Germany) interfaced with a ThermoFisher Scientific Orbitrap Fusion Lumos Tribrid mass spectrometer (Thermo Scientific, Bremen, Germany). Liquid chromatography was performed prior to MS/MS analysis for peptide separation. The HPLC column used is a Dionex 15 cm × 75 µm Acclaim Pepmap C18, 2 μm, 100 Å reversed-phase capillary chromatography column. 5 μL volumes of the peptide extract were injected and peptides eluted from the column by a 1100-min acetonitrile/0.1% formic acid gradient at a flow rate of 0.30 μL/min and introduced to the source of the mass spectrometer online. Nano electrospray ion source was operated at 2.3 kV. The digest was analyzed using the data-dependent multitask capability of the instrument acquiring full scan mass spectra using a Fourier transform (FT) orbitrap analyzer to determine peptide molecular weights and collision-induced dissociation (CID) MS/MS product ion spectra with an ion-trap analyzer to determine the amino acid sequence in successive instrument scans. The MS method used in this study was a data-dependent acquisition (DIA) with 3 s duty cycle. It includes one full scan at a resolution of 120,000 followed by as many MS/MS scans as possible on the most abundant ions in that full scan. Dynamic exclusion was enabled with a repeat count of 1 and ions within 10 ppm of the fragmented mass were excluded for 60 s. For the binding partner analysis, the data were analyzed using Sequest bundled into Proteome Discoverer V2.3. The database used to search the MS/MS spectra was the SwissProtKB mouse protein database containing 16,986 entries with an automatically generated decoy database (reversed sequences). The search was performed looking for fully tryptic peptides with a maximum of two missed cleavages. Oxidation of Methionine and acetylation of protein N-terminus were set as dynamic modifications and carbamidomethylation of Cysteine was set as static modifications. The precursor mass tolerance for these searches was set to 10 ppm and the fragment ion mass tolerance was set to 0.5 Da. Protein and peptide validation were performed using Scaffold (version 5.0.1, Proteome Software Inc., Portland, OR). Peptide identifications were accepted if they could be established at >5.0% probability to achieve an FDR <0.1% by the Peptide Prophet algorithm with Scaffold delta-mass correction. Protein identifications were accepted if they could be established at >5.0% probability to achieve an FDR <1.0% and contained at least 3 identified peptides. Protein probabilities were assigned by the Protein Prophet algorithm. Proteins that contained similar peptides and could not be differentiated based on MS/MS analysis alone were grouped to satisfy the principles of parsimony. Unique peptides including modified peptides were included, and semi-tryptic peptides were not considered. Dynamic exclusion was used, and the total number of spectra was counted. Internal normalization was performed using the spectral counts of IRAKM to control sample difference introduced in the immunoprecipitation and mass spectral experiments. Enrichment was assessed by comparing the normalized spectral counts of untreated and treated samples. Supplementary Data 1 included all the mitochondrial proteins with 2-fold increase in the treated versus the untreated sample. The mass spectrometry proteomics raw data have been deposited to the ProteomeXchange Consortium via the PRIDE partner repository with the dataset identifier PXD031934.

### Western blot

Tissues or cells were homogenized in NP-40 lysis buffer (﻿1% NP-40, 20 mM Tris-HCl pH 7.4, 150 mM NaCl, and EDTA-free protease inhibitors). The extract was centrifuged at 16,000 × *g* for 10 min at 4 °C. Supernatants were collected and analyzed with BCA (Thermo Scientific) to quantify protein concentration. Proteins were resolved by SDS-PAGE and transferred to PVDF membranes. Individual proteins were detected with specific antibodies and visualized on film using horseradish peroxidase-conjugated secondary antibodies and chemiluminescent substrate.

### siRNA and shRNA

For siRNA-mediated knockdown of mouse Slc25a1, siRNAs pool targeting Slc25a1 (Dharmacon, M-054849-00-0005, target sequences: 5′-GGTTTGGGATGTTCGAGTT-3′; 5′-GAAGCAGGGCTCAAACCAA-3′; 5′-GGCCAAAGGCATTCTACAA-3′; 5′-CCGAATACGTGAAGACGCA-3′) and non-targeting siRNA pool (Dharmacon, D-001206-13-05, target sequences: 5′-TAGCGACTAAACACATCAA-3′; 5′-TAAGGCTATGAAGAGATAC-3′; 5′-ATGTATTGGCCTGTATTAG-3′; 5′-ATGAACGTGAATTGCTCAA-3′) were transfected into adipocytes with the lipofectamine RNAiMAX reagent (ThermoFisher Scientific) according the manufacturer’s instruction. Forty-eight hours later, cells were harvested and subject to western blot analysis. For the rescue experiments in this study, shRNA targeting Slc25a1 lentivirus (Sigma-Aldrich, SHCLNG-NM_153150, target sequence: 5′-CTGCGGCTTGAAGATCCTAAA-3′) and non-target shRNA lentivirus (Sigma-Aldrich, SHC016, target sequence: 5′-GCGCGATAGCGCTAATAATTT-3′) were used for knockdown of Slc25a1 in stromal vascular fraction with puromycin selection followed by adipocytes differentiation.

### Reconstitution of IRAKM or Slc25a1 in adipocytes

Flag-tagged IRAKM WT, phos-mut (S167A/S170A), and mito-mut (R56A/K60A/K66A/R70A), or Slc25a1 WT and non-phosphorylated mutant (T155A) were cloned into pMXs-IP retrovirus vector. IRAKM KO or Slc25a1 KD stromal vascular fraction cells were transduced with viral supernatants to express Flag-tagged IRAKM or Slc25a1 followed by adipocytes differentiation.

### Chromatin immunoprecipitation

Chromatin immunoprecipitation was performed with Imprint® Chromatin Immunoprecipitation kit (Sigma-Aldrich, CHP1-96RXN). In brief, adipocytes were fixed with 1% formaldehyde for 10 min, followed by 0.125 M glycine quenching for 5 min. Adipocytes were scraped and lysed in nuclei preparation buffer and the DNA was fragmented by sonication. Immunoprecipitation was performed with 1 μg Pgc1a antibody overnight at 4 °C. Antibody-bound chromatins were reverse cross-linked and purified. The immunoprecipitated DNA was quantified by qPCR using QuantStudio 5 Real-Time PCR System.

### Real-time PCR

Total RNA was extracted with TRIzol (Invitrogen) and further purified using RNeasy Lipid Tissue Mini Kit (Qiagen) according to the manufacturer’s instructions. Then, 1 μg total RNA for each sample was reverse-transcribed using PrimeScript RT Reagent Kit from Takara. Delta-Delta Ct real-time PCR with Power SYBR Green PCR Master Mix (Life Technologies) and QuantStudio 5 Real-Time PCR System were used to analyze cDNA. Actin was used as endogenous control.

### Fatty acid synthesis analysis

Adipocytes differentiated from SVF cells were treated with IL-1β in presence of 2 μCi per ml ^14^C-glucose (PerkinElmer) for 24 h. Fatty acid synthesis was measured following the established protocol^57^. The cells were lysed with solution A (isopropyl alcohol:hexane:1N H_2_SO_4_ (v:v:v) = 4:1:1), and newly synthesize lipids were extracted with hexane. Hexane phase was dried, deacylated with ethanol:water:KOH (v:v:v) = 20:1:1 at 80 °C for an hour, neutralized with sulfuric acid, and dissolved the neutral FFAs in hexane. The samples were dried, reconstituted in scintillation fluid, and incorporation of ^14^C was determined by counting counts per minute. Fatty acid synthesis level was normalized to untreated group.

### Slc25a1 transport activity analysis

Transport assays with isolated mitochondria were performed following the established protocol^58^. Briefly, mitochondria were incubated in a buffer containing 100 mM KCl, 20 mM HEPES, pH 7.0, and 2 μg/ml rotenone. Transport at 9 °C was performed adding 1 mM of ^14^C-citrate (PerkinElmer) to mitochondria and terminated after 15 s by adding 20 mM BTA (benzene-1,2,3-tricarboxylate). Mitochondria were pelleted, suspended with water, and extracted with HClO_4_; and the extracts were used for scintillation counting of ^14^C. Slc25a1 transport activity was normalized to untreated group.

### OCR analysis

The OCR were measured using the Seahorse Extracellular Flux (XF24) Analyzer (Seahorse Bioscience). WT and IRAKM primary adipocytes were seeded at 1.5–2 × 10^4^ cells per Seahorse plate well, and stimulated with or without IL-1β (10 ng/ml). Three consecutive measurements were obtained in addition to those under basal conditions: (1) after the addition of 1.5 μM oligomycin; (2) after the sequential addition of 4.5 μM fluoro-carbonyl cyanide phenylhydrazone (FCCP); and (3) after the sequential addition of 1 μM rotenone plus 1 μM antimycin A. In this assay, maximum OCR occurs after the addition of FCCP. All OCR measures were performed three times in a mix–wait–measure cycle of 3, 2, and 3 min, respectively.

### Transmission electron microscopy

For ultrastructural analysis, cells were fixed for 1 h at 22 °C in 2% paraformaldehyde, 2.5% glutaraldehyde (Polysciences), and 0.05% malachite green (Sigma-Aldrich) in 100 mM sodium cacodylate buffer (pH 7.2). Malachite green was incorporated into the fixative for stabilization of lipid constituents soluble in aqueous glutaraldehyde. Samples were washed in cacodylate buffer and were post-fixed for 1 h in 1% osmium tetroxide (Polysciences). Samples were then rinsed extensively in distilled water before en bloc staining for 1 h with 1% aqueous uranyl acetate (Ted Pella). Following several rinses in distilled water, the samples were dehydrated in a graded series of ethanol and embedded in Eponate 12 resin (Ted Pella). Sections 95 nm in thickness were cut with a Leica Ultracut UC7 ultramicrotome (Leica Microsystems), then stained with uranyl acetate and lead citrate and viewed on a Tecnai G2 Spirit BioTWIN transmission electron microscope (FEI) at 60 kV.

### FAO assay

Mitochondrial fractions were isolated from WT and IRAKM KO adipocytes for immediate assay of FAO. The FAO rate was determined based on the oxidation of 1-^14^C palmitate (PerkinElmer)^20^. Complete oxidation was measured by the production of ^14^CO_2_, whereas incomplete oxidation was measured by the production of ^14^C-labeled acid-soluble metabolites. Total FAO was determined by the sum of complete and incomplete oxidation. The homogenate protein content was measured using a bicinchoninic acid assay (ThermoFisher Scientific).

### Citrate measurement

Mitochondria and cytoplasmic fractions were prepared as above. Metabolites were extracted from mitochondria and cytoplasmic fractions using methanol/water/chloroform. Metabolites were dried, and citrate was analyzed by liquid chromatography-mass spectrometry (LC-MS/MS) at Lerner Research Institute metabolomics core. A triple quadrupole mass spectrometer (Thermo Quantiva) was used for analysis of TCA compounds. The mass spectrometer was coupled to the outlet of an UHPLC system (Vanquish, Thermos Fisher Scientific, Waltham, MA, USA), including an autosampler with refrigerated sample compartment and inline vacuum degasser. A volume at 5 µl was injected onto a C18 column (Gemini, 3 µm, 2 × 150 mm, Phenomenex) for the separation of TCA compounds. Mobile phases were A (water containing 0.1% acetic acid and 5 mM ammonium acetate) and B (containing 0.1% acetic acid and 5 mM ammonium acetate). The run started with 0% B from 0 to 2 min at the flow rate of 0.3 ml/min. Solvent B was then increased linearly to 100% B from 2 to 8 min and held at 100% B from 8 to 18 min. The column was finally re-equilibrated with 0% B for 8 min. The HPLC eluent was directly injected into the triple quadrupole mass spectrometer and the TCA compounds were ionized using electrospray ionization (ESI) at negative mode with ESI spray voltage at 2.5 kV, sheath gas at 35 Arb and Aux gas at 20 Arb. The ion transfer tube and vaporizer temperatures were set at 350 °C and 250 °C, respectively. All the TCA compounds were analyzed using Selected Reaction Monitoring (SRM) and the SRM transitions (*m*/*z*) were their precursor to their product ions (*m*/*z*) as 87 > 43 for pyruvic acid, 115 > 71 for fumaric acid, 117 > 73 for succinic acid, 131 > 87 for oxaloacetic acid, 133 > 115 for malic acid, 145 > 101 for alpha-ketoglutaric acid, 173 > 77 for cis-aconitic acid, 191 > 111 for both citric and isocitric acids and 121 > 77 for succinic acid-d4 (internal standard). Ultrapure argon (99.99%) was used as a collision gas at the pressure of 2 millitorr for collision-induced dissociation. The peak areas for the TCA compounds and the internal standards are integrated using the software Xcalibur. Internal standard calibration curves were used for quantitation of TCA compounds in cell lysate samples. Citrate level was normalized to protein content.

### Fatty acid measurement

Adipose tissues were homogenized using cold 80% methanol/water mixture, and hydrolyzed by adding 2 mM BHT, 2 mM DTPA, 2 M NaOH and internal standard, mixed, and incubated at 60 °C for 2 h. All fatty acids were extracted with isopropyl alcohol/hexane/acetic acid buffer, dried, and analyzed by liquid chromatography-mass spectrometry (LC-MS/MS) at Lerner Research Institute metabolomics core. A triple quadrupole mass spectrometer (Thermo Quantiva) was used for analysis of fatty acid. The mass spectrometer was coupled to the outlet of an UHPLC system (Vanquish, Thermos Fisher Scientific, Waltham, MA, USA), including an autosampler with refrigerated sample compartment and inline vacuum degasser. A volume at 5 µl was injected onto a C18 column (Gemini, 3 µm, 2 × 150 mm, Phenomenex) for the separation of FA species. Mobile phases were A (water containing 0.1% acetic acid and 0.03% ammonium hydroxide) and B (methanol/acetonitrile (50/50) containing 0.1% acetic acid and 0.03% ammonium hydroxide). The run started with 75% mobile phase B from 0 to 2 min at the flow rate of 0.3 ml/min. Solvent B was then increased linearly to 100% B from 2 to 8 min and held at 100% B from 8 to 18 min. The column was finally re-equilibrated with 75% B for 8 min. The HPLC eluent was directly injected into the triple quadrupole mass spectrometer and the FA species were ionized using electrospray ionization at negative mode with ESI spray voltage at 2.5 kV, sheath gas at 35 Arb, and Aux gas at 20 Arb. The ion transfer tube and vaporizer temperatures were set at 350 °C and 250 °C, respectively. All the fatty acids were analyzed using Selected Reaction Monitoring (SRM) and the SRM transitions (*m*/*z*) were their precursor to precursor ions (*m*/*z*) as 255 > 255 for FA(16:0), 253 > 253 for FA(16:1), 283 > 283 for FA(18:0), 279 > 279 for FA(18:2), 277 > 277 for FA(18:3), 275 > 275 for FA(18:4), 311 > 311 for FA(20:0), 309 > 309 for FA(20:1), 307 > 307 for FA(20:2), 305 > 305 for FA(20:3), 303 > 303 for FA(20:4), 301 > 301 for FA(20:5), 339 > 339 for FA(22:0), 333 > 333 for FA(22:3), 331 > 331 for FA(22:4) 329 > 329 for FA(22:5), 327 > 327 for FA(22:6) and 315 > 315 for HPA (internal standard). Ultrapure argon (99.99%) was used as a collision gas at the pressure of 2 millitorr for collision-induced dissociation. Peak areas for all the FA species and the internal standard are integrated using the software Xcalibur. Internal standard calibration curves were used for quantitation of FA species in cell lysate samples. Fatty acid level was normalized to weight of adipose tissues.

### Triglyceride measurement

Tissue triglyceride was measured with Triglyceride Quantification Colorimetric kit (Sigma-Aldrich, MAK266-1KT). Tissues were dissected, and homogenized in 5% NP-40 Substitute buffer. Triglyceride was measured according to the manufacturer’s instruction. Triglyceride level was normalized to weight of adipose tissues.

### Acetyl-CoA measurement

Acetyl-CoA in adipocytes or adipose tissues was measured with PicoProbe™Acetyl-CoA Fluorometric Assay kit (BioVision, K317). Cells were deproteinized using a perchloric acid/KOH protocol (BioVision, K808). Acetyl-CoA was measured according to the manufacturer’s instruction. Acetyl-CoA level was normalized to untreated group.

### Indirect calorimetry analysis and EchoMRI

HFD-fed or chow diet-fed mice were allowed to equilibrate to metabolic cage environments for ~24 h before entering into 24-h periods at thermoneutrality (30 °C), room temperature (22 °C) or cold temperature (4 °C). Oxygen consumption (VO_2_), carbon dioxide production (VCO_2_), heat production, and the respiratory exchange ratio were constantly monitored using the Oxymax CLAMS system (Columbus Instruments). The data were normalized to total body weight. Fat mass and lean mass were measured using an EchoMRI-100H system (EchoMRI).

### Glucose tolerance test (GTT), insulin tolerance test (ITT), and body temperature measurement

For the GTT, mice were fasted overnight and glucose (2.5 g per kg body weight) was injected peritoneally. For the ITT, mice were fasted for 6 h and insulin (0.75 U per kg body weight) was injected peritoneally. Blood glucose levels were measured using an AimStrip Plus Blood Glucose Testing System (Germaine Laboratories). Rectal temperature was measured using a MicroTherma 2T Hand Held Thermometer with a rectal probe (Braintree Scientific).

### Statistics

All data are shown as mean ± s.e.m. Replicates are indicated in figure legends. Statistical analysis was carried out with GraphPad Prism 8 software. When comparing two groups, statistical analysis was performed using a two-tailed Student’s *t*-test. For analysis of more than two groups, analysis of variance (ANOVA) Tukey-Krammer post hoc analysis was used to determine equality of variance. In all tests, *p* values <0.05 were considered statistically significant.

### Reporting summary

Further information on research design is available in the Nature Research Reporting Summary linked to this article.

## Supplementary information

Supplementary Information

Description of Additional Supplementary Information

Supplementary Data

Reporting Summary

## Data Availability

The authors declare that all data supporting this study’s findings are available within this paper and its Supplementary and Source Files. The mass spectrometry proteomics data have been deposited to the ProteomeXchange Consortium via the PRIDE partner repository with the dataset identifier PXD031934 (Hyperlink). All datasets generated during and/or analyzed during the current study are also available from the corresponding authors on reasonable request. Source data are provided with this paper.

## Source data

Source Data
