# Supplementary material for: IL-1R-IRAKM-Slc25a1 signaling axis reprograms lipogenesis in adipocytes to promote diet-induced obesity in mice
Source: Nat Commun. 2022 May 18;13:2748. doi: 10.1038/s41467-022-30470-w (PMC9117277; doi:10.1038/s41467-022-30470-w)

Supplementary Information

IL-1R-IRAKM-Slc25a1 signaling axis reprograms lipogenesis in adipocytes to promote diet-induced obesity in mice

Weiwei Liu[1], Hao Zhou[1,5], Han Wang[1], Quanri Zhang[1], Renliang Zhang[2], Belinda Willard[2], Caini Liu[1], Zizhen Kang[3], Xiao Li[4]*, Xiaoxia Li[1]*

[1]Department of Inflammation and Immunity, Lerner Research Institute, Cleveland Clinic, Cleveland, OH 44106, USA.
[2]Proteomics and Metabolomics Core, Lerner Research Institute, Cleveland Clinic, Cleveland, OH 44106, USA.
[3]Department of Pathology, Carver College of Medicine, University of Iowa, Iowa City, IA 52242, USA.
[4]Center for RNA Science and Therapeutics, School of Medicine, Case Western Reserve University, Cleveland, OH 44106, USA
[5]Present address: Division of Transplant Surgery, Department of Surgery, Brigham and Women's Hospital, Harvard Medical School, Boston, MA 02115, USA.

*Correspondence: xiao.li9@case.edu ; lix@ccf.org

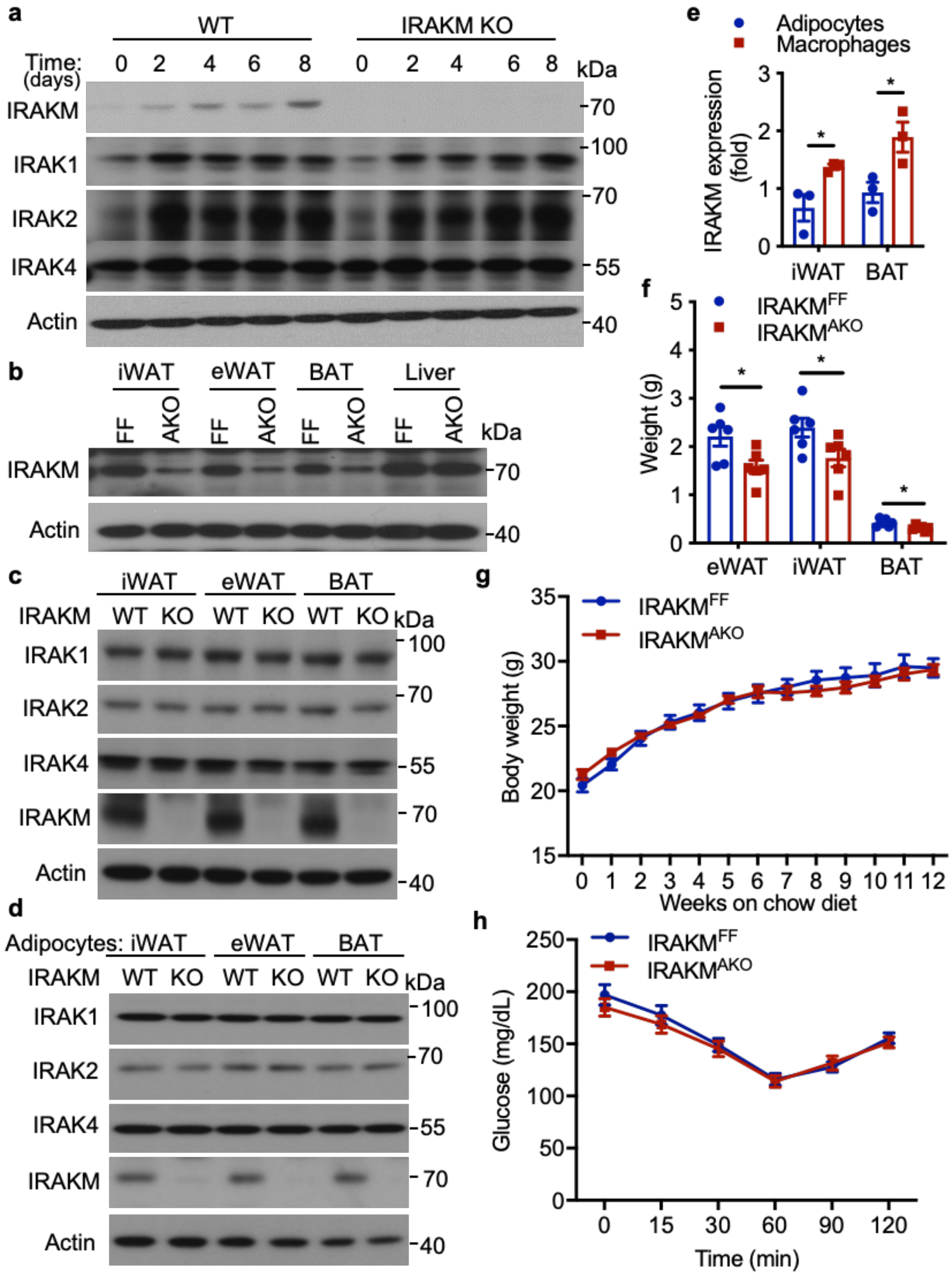
a
WT
IRAKM KO
Time: (days)
0 2 4 6 8 0 2 4 6 8
kDa
IRAKM
IRAK1
IRAK2
IRAK4
Actin
70
100
70
55
40
b
iWAT eWAT BAT Liver
FF AKO FF AKO FF AKO FF AKO
kDa
IRAKM
Actin
70
40
c
iWAT eWAT BAT
IRAKM WT KO WT KO WT KO
kDa
IRAK1
IRAK2
IRAK4
IRAKM
Actin
100
70
55
70
40
d
Adipocytes: iWAT eWAT BAT
IRAKM WT KO WT KO WT KO
kDa
IRAK1
IRAK2
IRAK4
IRAKM
Actin
100
70
55
70
40
e
Adipocytes
Macrophages
IRAKM expression (fold)
iWAT BAT
f
IRAKM^FF
IRAKM^AKO
Weight (g)
eWAT iWAT BAT
g
IRAKM^FF
IRAKM^AKO
Body weight (g)
Weeks on chow diet
h
IRAKM^FF
IRAKM^AKO
Glucose (mg/dL)
Time (min)

Supplementary Fig. 1 IRAKM was expressed in adipose tissues. **a**, Western blot analysis of IRAKs expression during primary adipocytes differentiation from stromal vascular fraction of iWAT from WT and IRAKM KO mice. Data are representative of three independent experiments. **b**, Western blot analysis of IRAKM expression in iWAT, eWAT, BAT and liver from $IRAKM^{F/F}$ and $IRAKM^{AKO}$ mice. Data are representative of two independent experiments. **c**, Western blot analysis of IRAKs expression in iWAT, eWAT, and BAT from WT and IRAKM KO mice. Data are representative of two independent experiments. **d**, Western blot analysis of IRAKs expression in isolated adipocytes of iWAT, eWAT, and BAT from WT and IRAKM KO mice. Data are representative of two independent experiments. **e**, IRAKM expression in mature adipocytes and $F4/80^{+}$ macrophages of iWAT and BAT tissues were analyzed by real-time PCR (n=3; from left to right: P=0.038, 0.038). **f**, adipose tissues weight of HFD-fed $IRAKM^{F/F}$ and $IRAKM^{AKO}$ mice (n = 6 males per group; from left to right: P=0.025, 0.039, 0.021). **g**, Body weight of $IRAKM^{F/F}$ and $IRAKM^{AKO}$ mice fed with chow diet for 11 weeks (n = 6 males per group). **h**, Insulin tolerance test (ITT) were analyzed in chow diet-fed $IRAKM^{F/F}$ and $IRAKM^{AKO}$ mice (n=6 mice for each group). Statistical significance was determined by two-tailed Student's t-test (**e**-**f**). *P < 0.05. All data represent mean ± s.e.m. Source data are provided as the Source Data file.

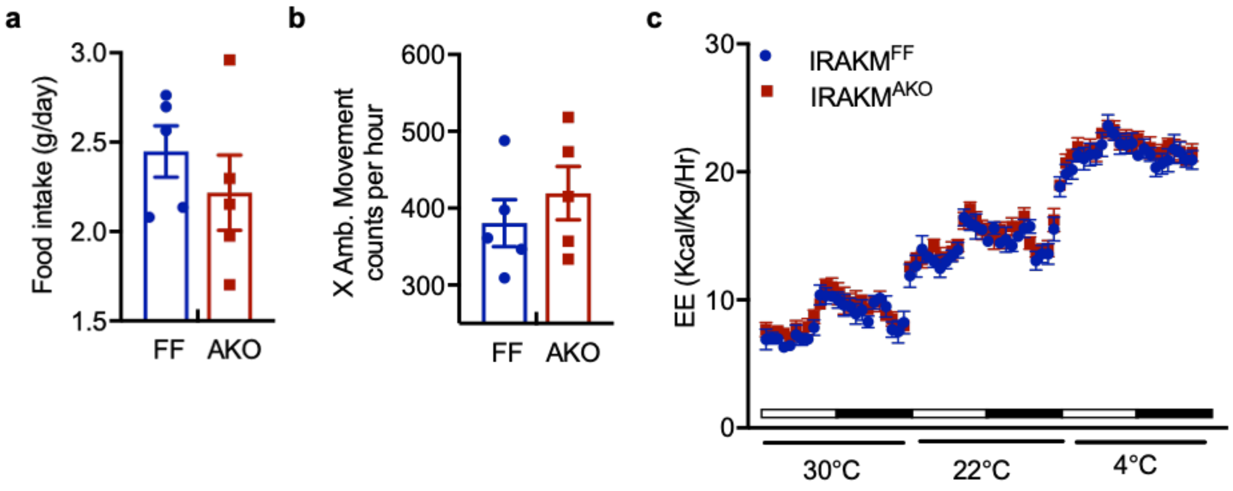

Supplementary Fig. 2 Adipocyte-specific IRAKM deficiency promotes energy expenditure. **a-b**, Food intake (**a**) and physical activity (**b**) of HFD-fed IRAKM$^{F/F}$ and IRAKM^{AKO} mice were analyzed by Columbus Oxymax metabolic chambers (n=5 mice for each group). **c**, energy expenditure (EE) of chow diet-fed IRAKM$^{F/F}$ and IRAKM^{AKO} mice were analyzed by Columbus Oxymax metabolic chambers (n=5 mice for each group). Statistical significance was determined by two-tailed Student's t-test (**a**, **b**), or two-way ANOVA (**c**). However, there was no significance between comparison. All data represent mean ± s.e.m. Source data are provided as the Source Data file.

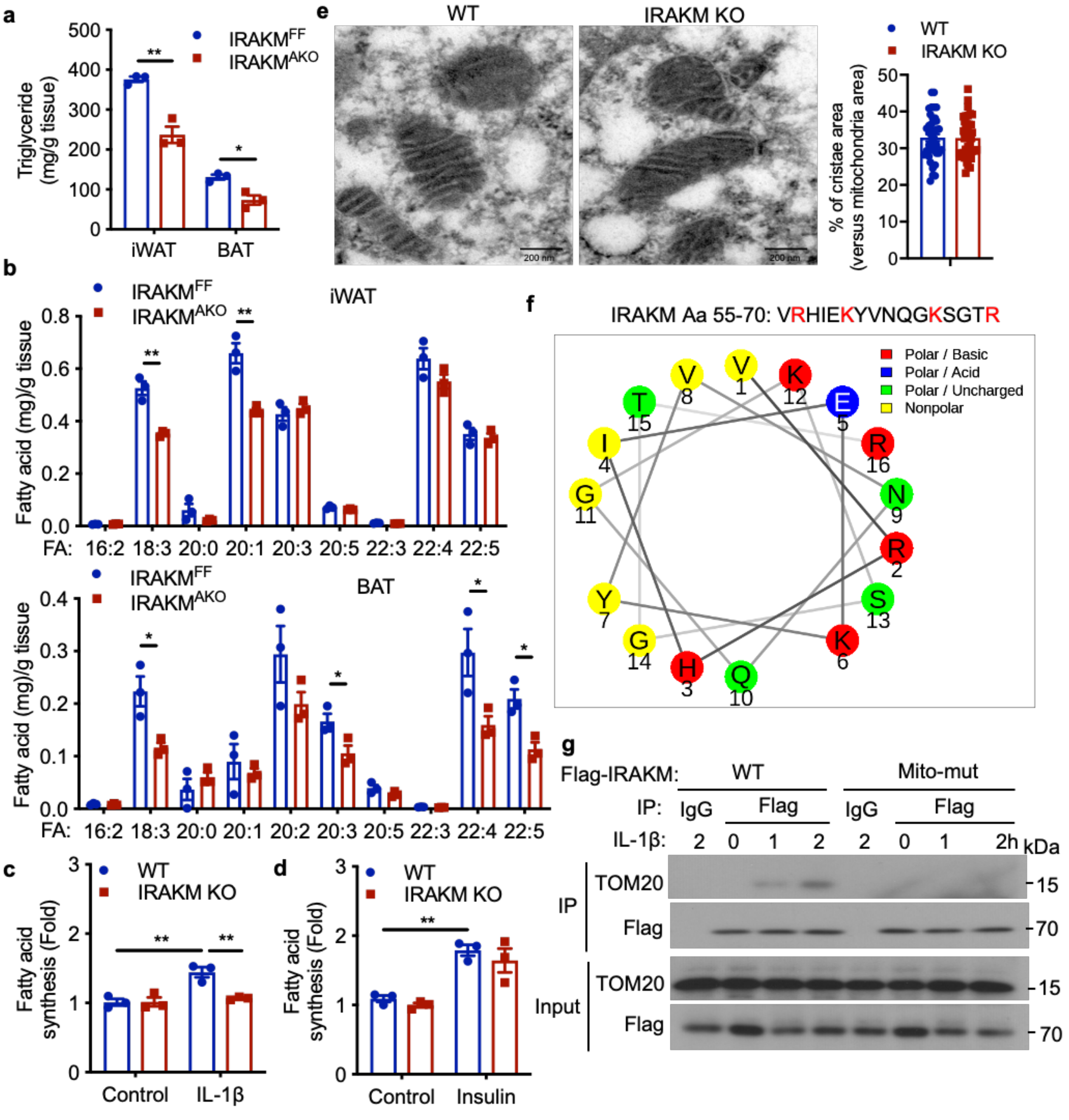
a
Triglyceride (mg/g tissue)
500
400
300
200
100
0
IRAKMFF
IRAKMAKO
**
*
iWAT
BAT
b
IRAKMFF
IRAKMAKO
iWAT
Fatty acid (mg)/g tissue
0.8
0.6
0.4
0.2
0.0
FA: 16:2 18:3 20:0 20:1 20:3 20:5 22:3 22:4 22:5
BAT
0.4
0.3
0.2
0.1
0.0
FA: 16:2 18:3 20:0 20:1 20:2 20:3 20:5 22:3 22:4 22:5
c
Fatty acid synthesis (Fold)
WT
IRAKM KO
Control
IL-1β
d
WT
IRAKM KO
Control
Insulin
e
WT
IRAKM KO
200 nm
% of cristae area (versus mitochondria area)
50
40
30
20
10
0
f
IRAKM Aa 55-70: VRHIEKYVNQGKSGTR
Polar / Basic
Polar / Acid
Polar / Uncharged
Nonpolar
V 1
R 2
H 3
I 4
E 5
K 6
Y 7
V 8
N 9
Q 10
G 11
K 12
S 13
G 14
T 15
R 16
g
Flag-IRAKM:
WT
Mito-mut
IP:
IgG
Flag
IL-1β:
2 0 1 2 2 0 1 2h
kDa
IP
TOM20
Flag
Input
TOM20
Flag
15
70
15
70

Supplementary Fig. 3 IL-1β induces translocation of IRAKM into mitochondria to promote de novo fatty acid synthesis. **a**, Triglycerides were analyzed in iWAT and BAT from IRAKM$^{F/F}$ and IRAKM^{AKO} mice after HFD feeding (n=3; from left to right: P=0.003, 0.012). **b**, Levels of total fatty acids in iWAT and BAT from IRAKM$^{F/F}$ and IRAKM^{AKO} mice after HFD feeding were detected with LC-MS analysis (n=3; from left to right: iWAT: P=0.002, 0.005; BAT: P=0.022, 0.043, 0.044, 0.013). **c**, *de novo* fatty acid synthesis measured by the conversion of ^{14}C-glucose to lipids in brown adipocytes from WT and IRAKM KO mice treated with or without IL-1β for 24h (n=3; from left to right: P=0.0075, 0.0069). **d**, *de novo* fatty acid synthesis measured by the conversion of ^{14}C-glucose to lipids in primary white adipocytes from WT and IRAKM KO mice treated with or without insulin for 24h (n=3; P=0.0016). **e**, Left: representative transmission electron microscopy analysis of primary WT and IRAKM KO adipocytes (5 views per group, n=3) . Right: morphometric analysis of cristae area versus mitochondria area in 40 randomly selected mitochondria per group. **f**, Secondary helical wheel structure of IRAKM potential mitochondrial localization signal (MLS) peptide at the N-terminal generated by http://lbqp.unb.br/NetWheels/. **g**, Flag-tagged wild-type and IRAKM mito-mutant were restored in IRAKM KO primary adipocytes. Co-immunoprecipitation (IP) analysis was performed with anti-Flag antibody in whole cell lysates of IL-1β treated primary adipocytes and followed by western blot analysis for indicated proteins. Data are representative of two independent experiments. Statistical significance was determined by two-tailed Student's t-test (**a-d**). *P < 0.05. **P < 0.01. All data represent mean ± s.e.m. Source data are provided as the Source Data file.

a

Protein	Accession	Peptides	Coverage
Slc25a1	Q8JZU2	6	21%
IRAKM	Q8K4B2	10	21%
Il1r1	P13504	11	30%
MyD88	P22366	13	50%
IRAK4	Q8R4K2	3	7.2%

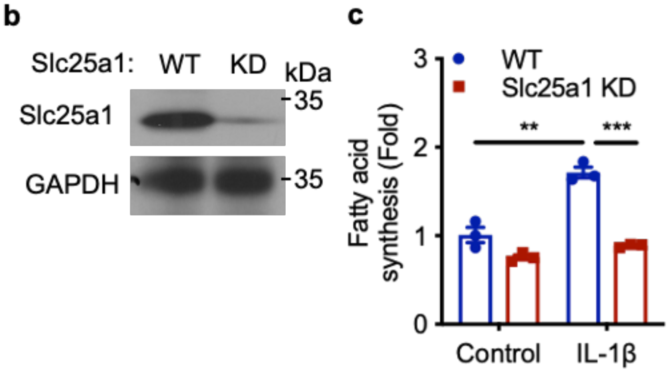

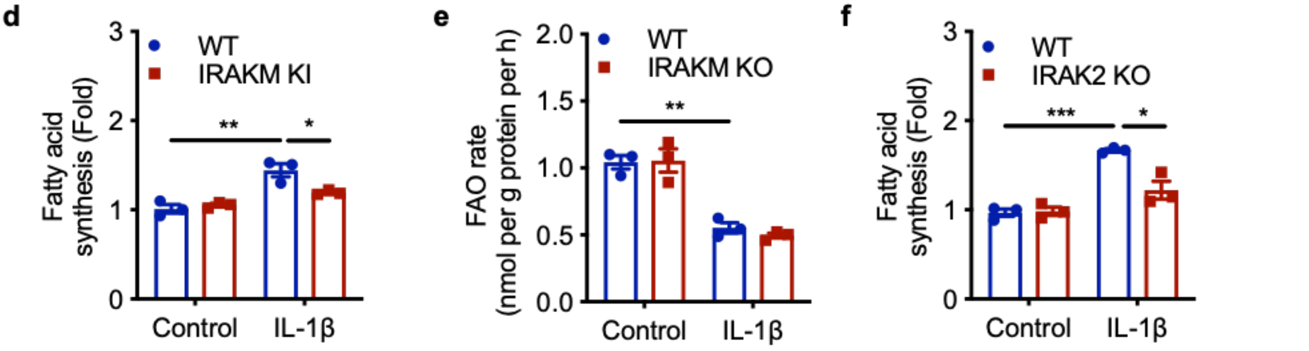

Supplementary Fig. 4 IRAKM promotes IL-1β induced mitochondrial citrate transport via interaction with Slc25a1. **a**, Proteomic analysis identifying Slc25a1 among the proteins immunoprecipitated from adipocytes stimulated with IL-1β for 1 hour using an anti-IRAKM antibody. **b**, Slc25a1 expression in WT and Slc25a1 knockdown(KD) adipocytes. Data are representative of two independent experiments. **c**, *de novo* fatty acid synthesis measured by the conversion of ^{14}C-glucose to lipids in primary WT and Slc25a1 KD adipocytes treated with or without IL-1β for 24h (n=3; from left to right: P=0.0026, 0.00019). **d**, *de novo* fatty acid synthesis measured by the conversion of ^{14}C-glucose to lipids in primary WT and IRAKM KI brown adipocytes treated with or without IL-1β for 24h (n=3; from left to right: P=0.0075, 0.027). **e**, [1-^{14}C]-palmitic acid oxidation rate in mitochondria from wild-type and IRAKM KO primary white adipocytes treated with or without IL-1β for 24h (n=3; P=0.0015). **f**, *de novo* fatty acid synthesis measured by the conversion of ^{14}C-glucose to lipids in primary WT and IRAK2 KO white adipocytes treated with or without IL-1β for 24h (n=3; from left to right: P=0.000095, 0.012). Statistical significance was determined by two-tailed Student's t-test (**c**-**f**). *P < 0.05. **P < 0.01. ***P < 0.001. All data represent mean ± s.e.m. Source data are provided as the Source Data file.

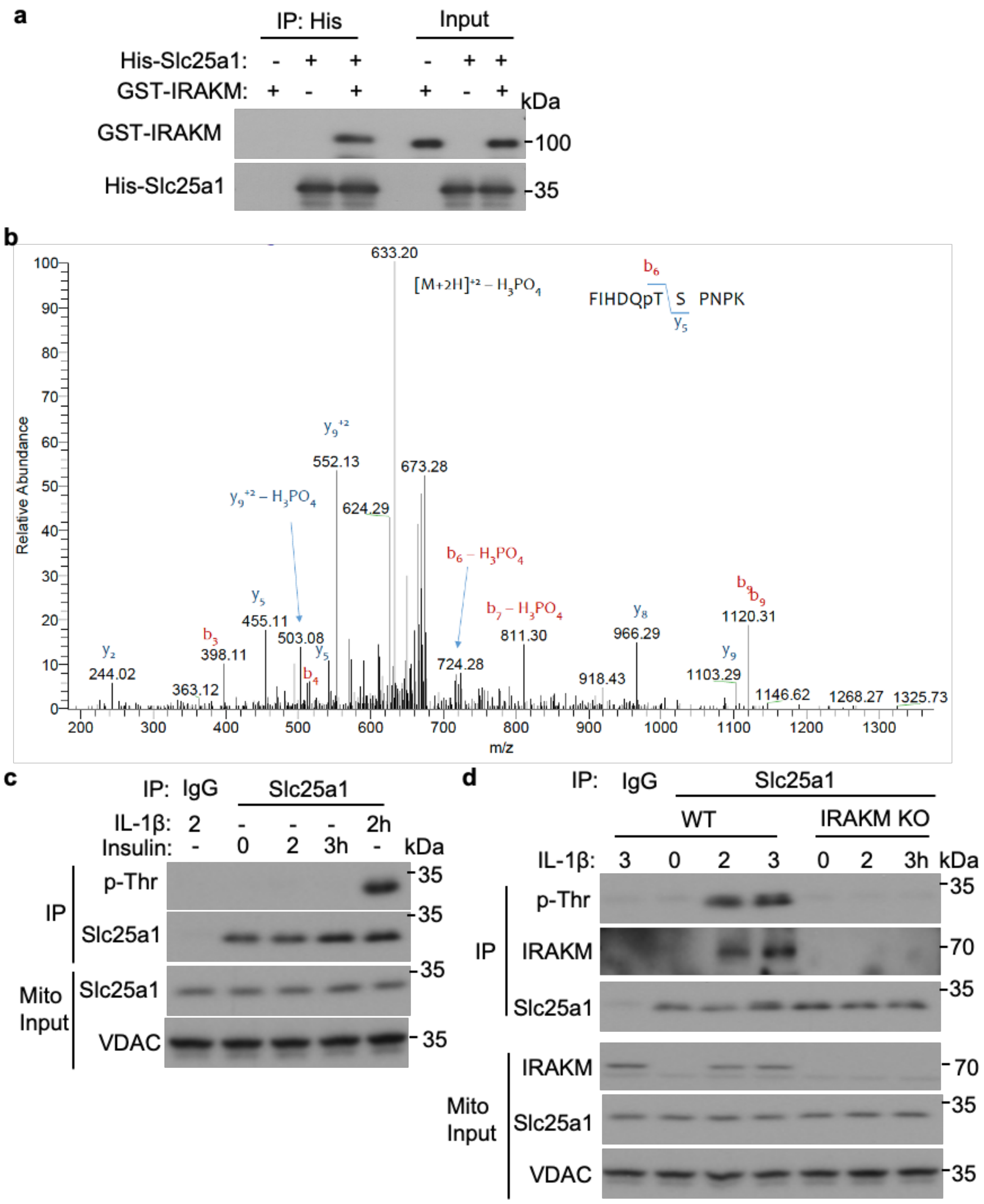

Supplementary Fig. 5 IRAKM as a novel kinase phosphorylates Slc25a1 to regulate mitochondrial citrate transport. **a**, Recombinant His-tagged Slc25a1 and GST-tagged IRAKM protein were combined and followed by immunoprecipitation (IP) with anti-His antibody and analyzed by Western blot analysis with indicated antibodies. Data are representative of three independent experiments. **b**, the fragment ion mass spectrum depicting the phosphopeptide showing Slc25a1Thr155 phosphorylation. **c**, Co-immunoprecipitation (IP) analysis was performed with anti-Slc25a1 antibody in mitochondrial lysate of IL-1β or insulin treated primary adipocytes and followed by western blot analysis for indicated proteins. Data are representative of two independent experiments. **d**, Co-IP analysis was performed with anti-Slc25a1 antibody in mitochondrial lysate of IL-1β treated primary WT and IRAKM KO brown adipocytes and followed by western blot analysis for indicated proteins. Data are representative of two independent experiments. Source data are provided as the Source Data file.

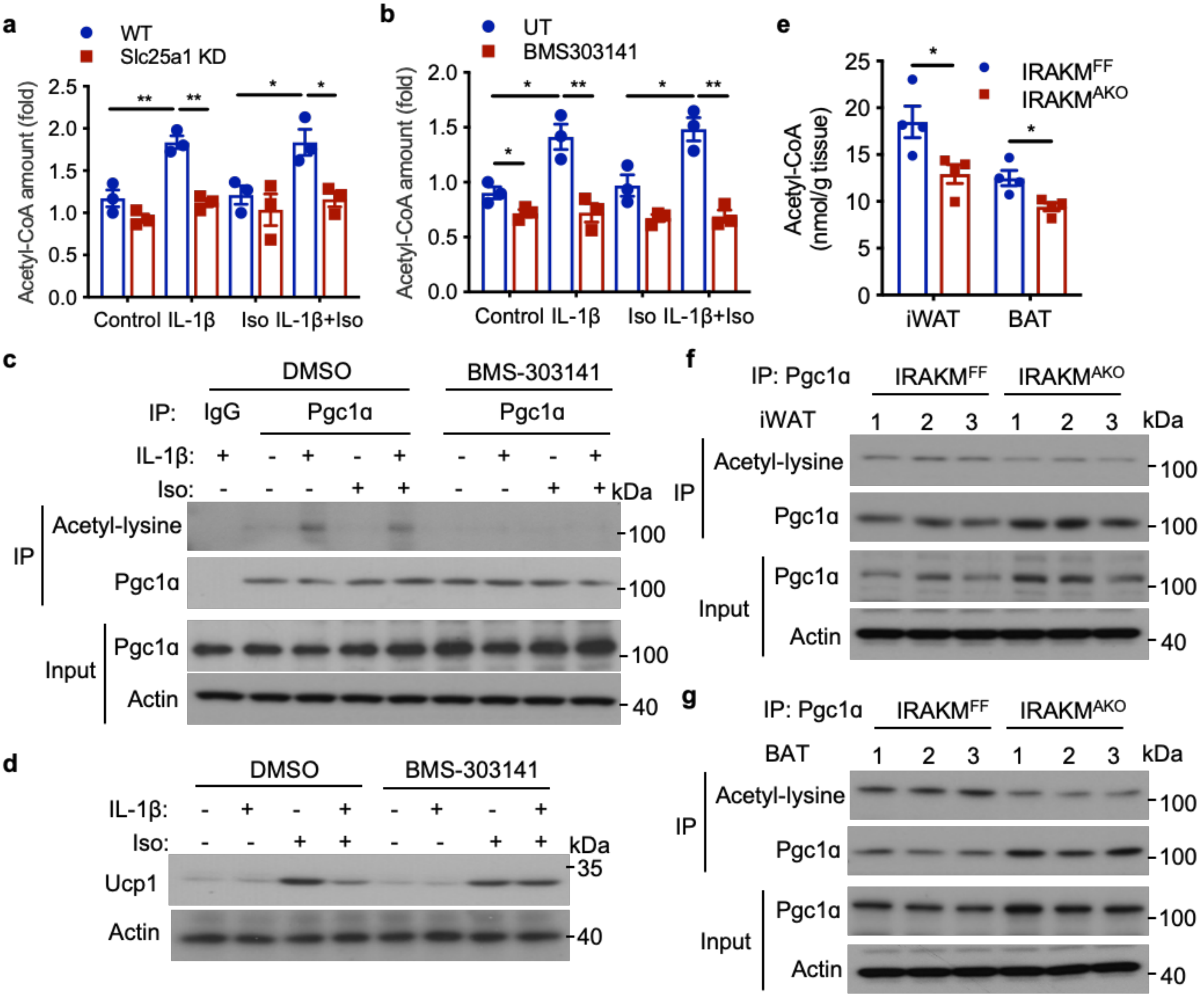

Supplementary Fig. 6 IRAKM is required for IL-1β induced Pgc1a acetylation to regulate Ucp1 expression. **a**, Acetyl-CoA levels in IL-1β pre-treated primary WT and Slc25a1 knockdown(KD) adipocytes followed by Isoproterenol stimulation for 24h (n=3; from left to right: P=0.0062, 0.0013, 0.0089, 0.027). **b**, Acetyl-CoA levels in IL-1β pre-treated primary adipocytes in presence of ACLY inhibitor BMS-303141 (20μM) followed by Isoproterenol stimulation for 24h (n=3; from left to right: P=0.045, 0.016, 0.0083, 0.023, 0.0026). **c**, Co-immunoprecipitation (IP) was performed with anti-Pgc1a antibody in cell lysate of IL-1β pre-treated primary adipocytes in presence of ACLY inhibitor BMS-303141 followed by Isoproterenol stimulation for 24h and analyzed by western blot for indicated proteins. Data are representative of two independent experiments. **d**, Primary adipocytes were pre-treated with IL-1β in presence of ACLY inhibitor BMS-303141 for 2h, and then stimulated with Isoproterenol for 24h. Western blot analysis was performed for indicated proteins. Data are representative of two independent experiments. **e**, Acetyl-CoA levels in iWAT and BAT tissues from HFD-fed IRAKM$^{F/F}$ and IRAKM^{AKO} mice (n=4; from left to right: P=0.029, 0.016). **f-g**, Co-IP was performed with anti-Pgc1a antibody in cell lysate of iWAT (**f**) and BAT (**g**) tissues from HFD-fed IRAKM$^{F/F}$ and IRAKM^{AKO} mice and analyzed by western blot for indicated proteins. Data are representative of two independent experiments. Statistical significance was determined by two-tailed Student's t-test (**a-b**, **e**). *P < 0.05. **P < 0.01. All data represent mean ± s.e.m. Source data are provided as the Source Data file.

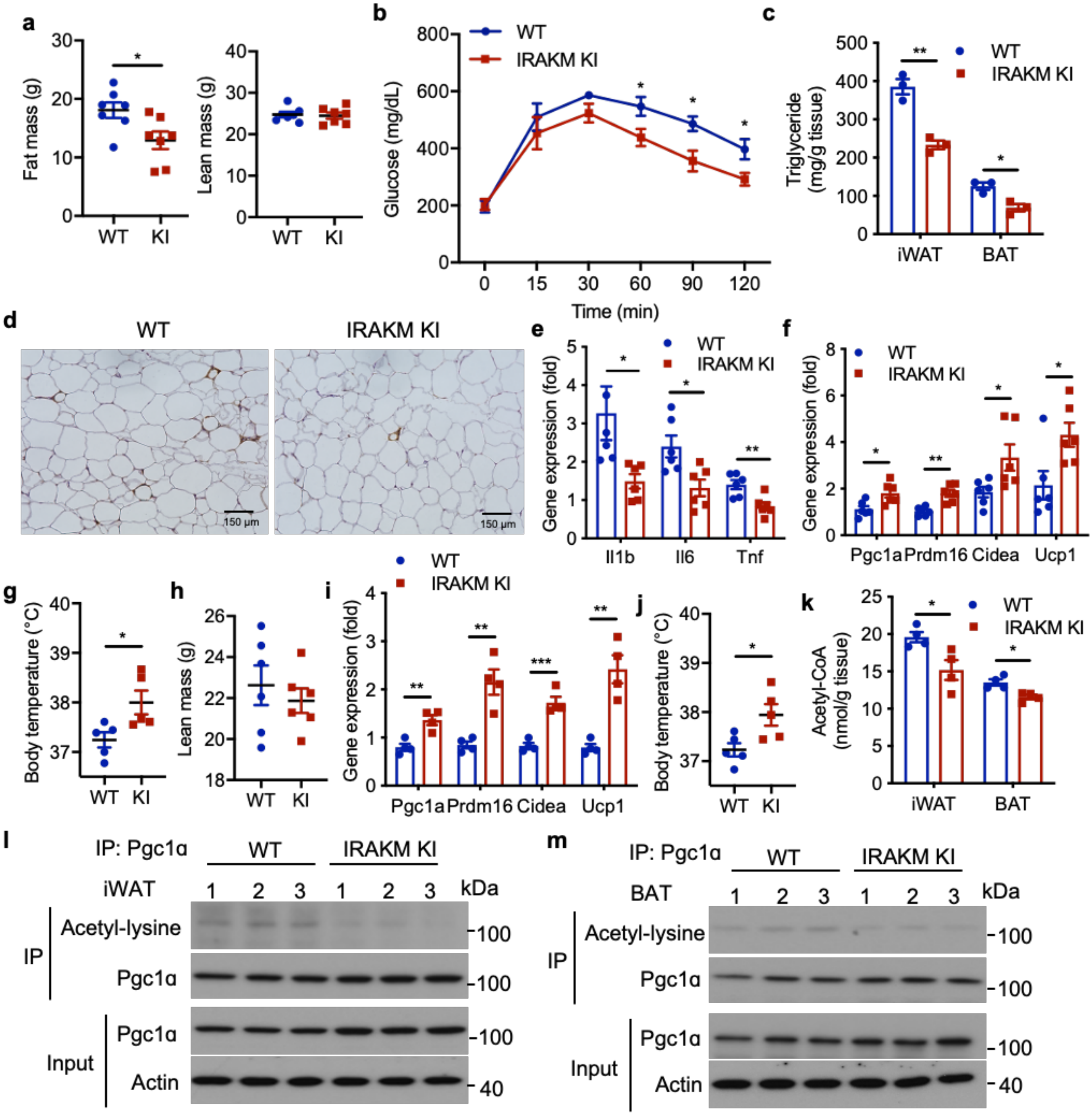
a
Fat mass (g)
Lean mass (g)
WT
KI
b
WT
IRAKM KI
Glucose (mg/dL)
Time (min)
c
Triglyceride (mg/g tissue)
iWAT
BAT
d
WT
IRAKM KI
150 μm
e
Gene expression (fold)
Il1b
Il6
Tnf
f
Pgc1a
Prdm16
Cidea
Ucp1
g
Body temperature (°C)
h
Lean mass (g)
i
j
k
Acetyl-CoA (nmol/g tissue)
l
IP: Pgc1α
iWAT
kDa
IP
Acetyl-lysine
Pgc1α
Input
Actin
m
BAT

Supplementary Fig. 7 IRAKM kinase-dead KI mice are protected against obesity. **a**, EchoMRI analysis of WT and IRAKM KI mice after HFD feeding (n=7 mice for each group; P=0.024). **b**, Glucose tolerance test (GTT) were analyzed in HFD-fed WT and IRAKM KI mice (n=7 mice for each group; from left to right: P=0.03, 0.012, 0.024). **c**, Triglycerides were analyzed in iWAT and BAT from WT and IRAKM KI mice after HFD feeding (n=3 mice for each group; from left to right: P=0.0026, 0.014). **d**, representative Mac2 staining of iWAT sections from HFD-fed WT and IRAKM KI mice (3 views per slide and 3 sections per mouse, n=5 mice per group). e, Inflammatory genes expression in iWAT of HFD-fed WT and IRAKM KI mice were analyzed by real-time PCR (n=6; from left to right: P=0.033, 0.013, 0.005). **f**, Thermogenic genes expression in iWAT of HFD-fed WT and IRAKM KI mice were analyzed by real-time PCR (n=6; from left to right: P=0.013, 0.0012, 0.034, 0.021). **g**, Rectal temperatures for HFD-fed WT and IRAKM KI mice (n=5 mice for each group; P=0.029). **h**, EchoMRI analysis of lean mass from WT and IRAKM KI mice after ZFD feeding (n=6 mice for each group). **i**, Thermogenic genes expression in iWAT of ZFD-fed WT and IRAKM KI mice were analyzed by real-time PCR (n=4; from left to right: P=0.004, 0.003, 0.0005, 0.0016). **j**, Rectal temperatures for ZFD-fed WT and IRAKM KI mice (n=5 mice for each group; P=0.025). **k**, Acetyl-CoA levels in iWAT and BAT tissues from HFD-fed WT and IRAKM KI mice (n=4; from left to right: P=0.026, 0.01). **l-m**, Co-IP was performed with anti-Pgc1a antibody in cell lysate of iWAT (**l**) and BAT (**m**) tissues from HFD-fed WT and IRAKM KI mice and analyzed by western blot for indicated proteins. Data are representative of two independent experiments. Statistical significance was determined by two-tailed Student's t-test (**a-c, e-k**). *P < 0.05. **P < 0.01. ***P < 0.001. All data represent mean ± s.e.m. Source data are provided as the Source Data file.

Supplementary Table 1. Primers for Real-time PCR

Gene	Forward Primer (5' to 3')	Reverse Primer (5' to 3')
Ucp1	TGTAAACAACAAAATACTGGCAGATG	GACCCGAGTCGCAGAAAAG
Pgc1a	TATGGAGTGACATAGAGTGTGCT	CCACTTCAATCCACCCAGAAAG
Cidea	TGACATTCATGGGATTGCAGAC	GGCCAGTTGTGATGACTAAGAC
Prdm16	CCCCACATTCCGCTGTGAT	CTCGCAATCCTTGCACTCA
Il1b	GCAGCAGCACATCAACAA	CACGGGAAAGACACAGGTA
Il6	GGACCAAGACCATCCAATTC	ACCACAGTGAGGAATGTCCA
Tnf	CAAAGGGAGAGTGGTCAGGT	ATTGCACCTCAGGGAAGAAT
Actin	GGTCATCACTATTGGCAACG	ACGGATGTCAACGTCACACT
Ucp1 for ChIP	CAAA TGGTGACCGGGTGCCCT	GGGTGACTGACCCTCTGTGACG

Uncropped blots

Supplementary Fig. 1a

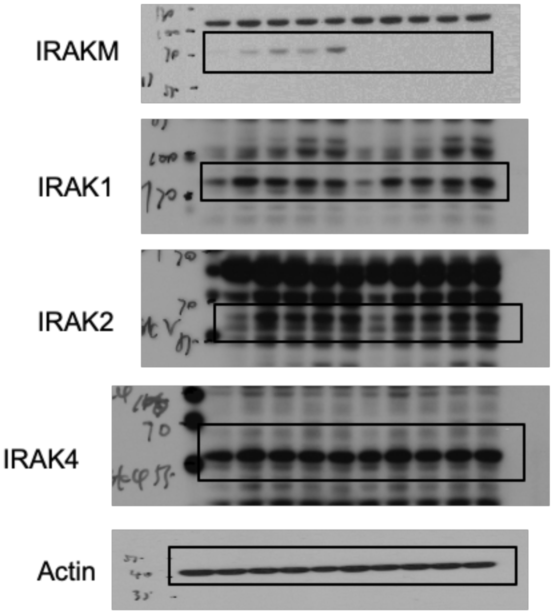

Supplementary Fig. 1b

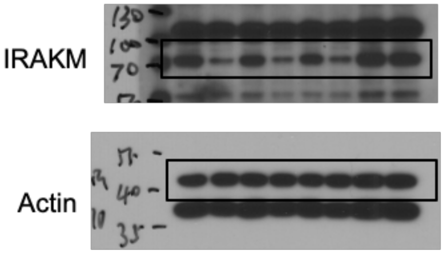

Supplementary Fig. 1c

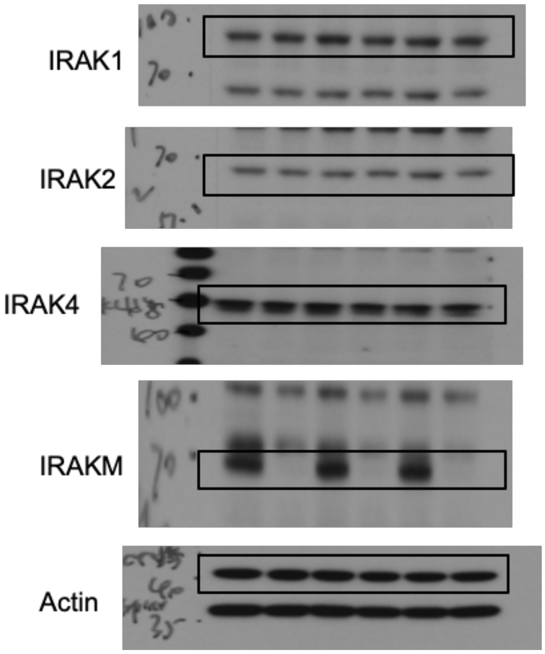

Supplementary Fig. 1d

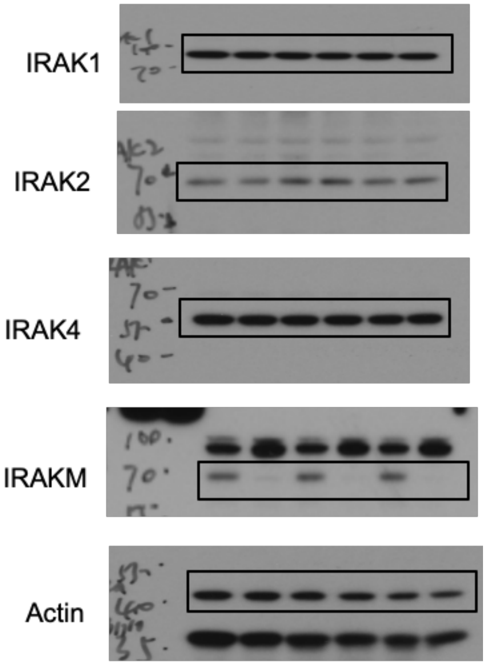

Uncropped blots

Supplementary Fig. 3g

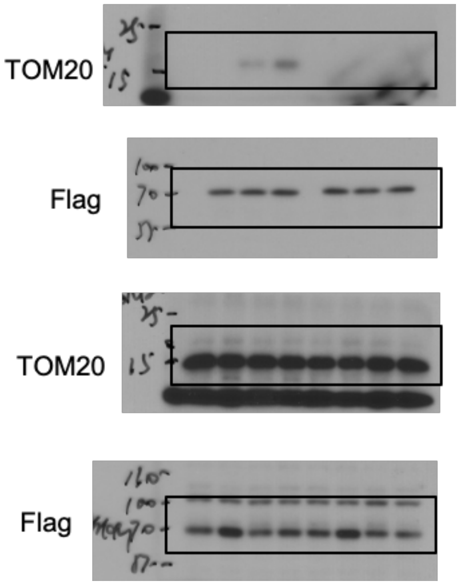

Uncropped blots

Supplementary Fig. 4b

Slc25a1

GAPDH

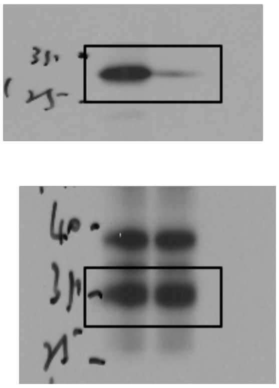

Uncropped blots

Supplementary Fig. 5a

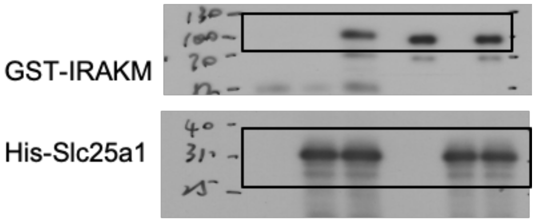

Supplementary Fig. 5c

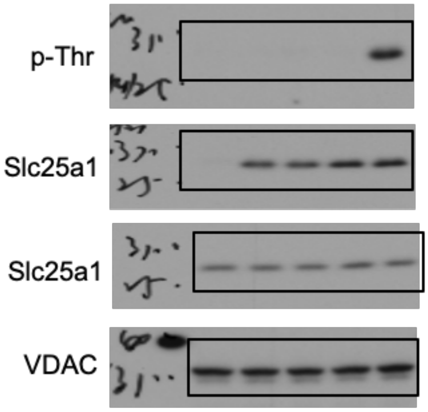

Supplementary Fig. 5d

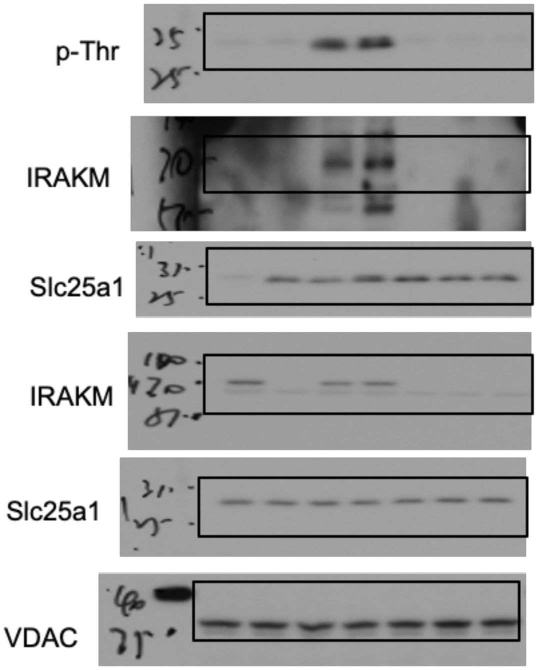

Uncropped blots

Supplementary Fig. 6c

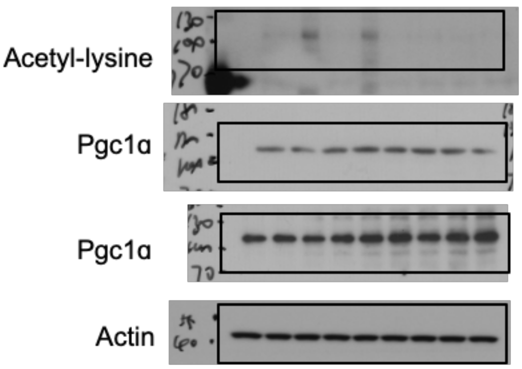

Supplementary Fig. 6d

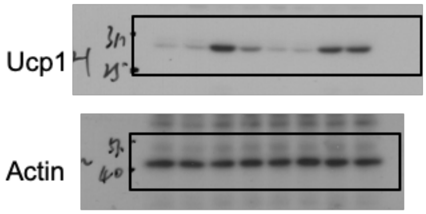

Supplementary Fig. 6f

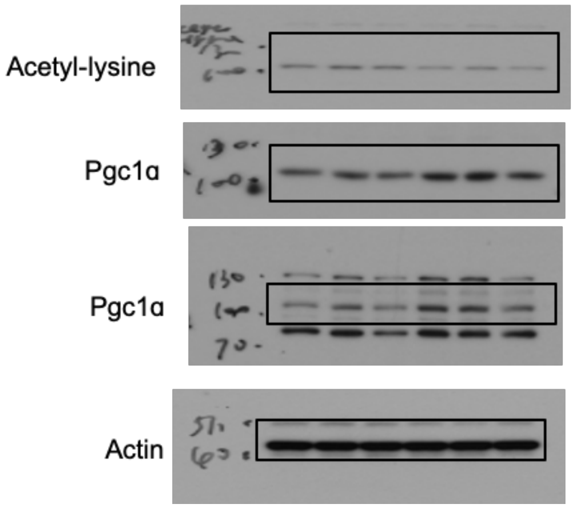

Supplementary Fig. 6g

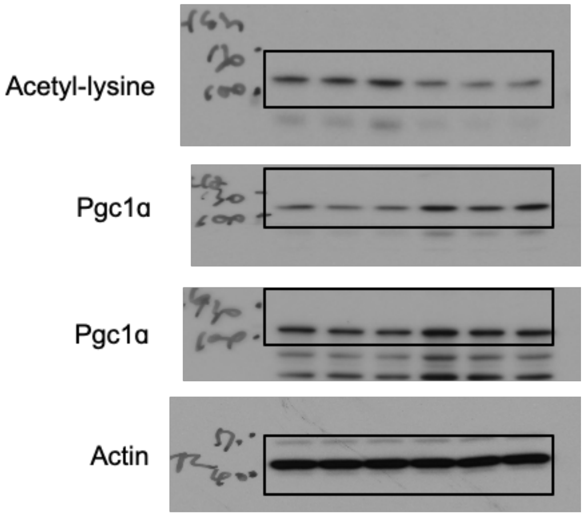

Uncropped blots

Supplementary Fig. 7l

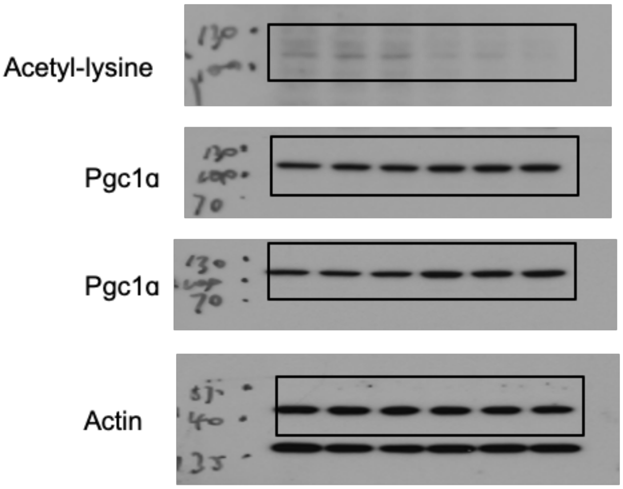

Supplementary Fig. 7m

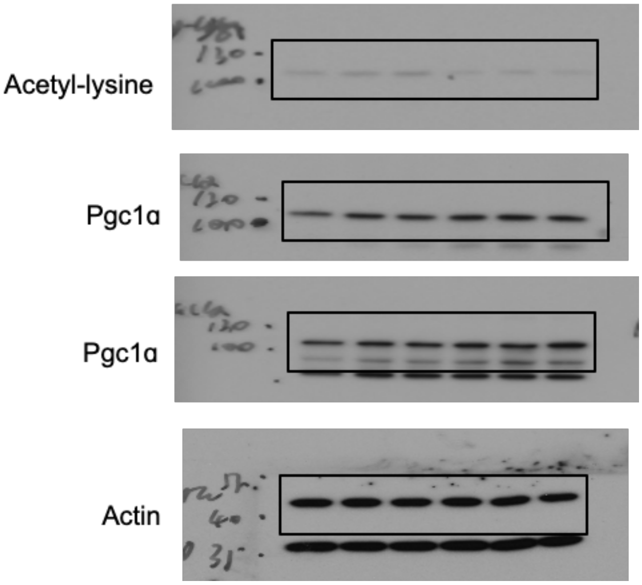

Supplement: Supplementary file 1 — Supplementary Information [file 41467_2022_30470_MOESM1_ESM.pdf]
