# Supplementary material for: IL-1R-IRAKM-Slc25a1 signaling axis reprograms lipogenesis in adipocytes to promote diet-induced obesity in mice
Source: Nat Commun. 2022 May 18;13:2748. doi: 10.1038/s41467-022-30470-w (PMC9117277; doi:10.1038/s41467-022-30470-w)
Supplement: Supplementary file 2 — Description of Additional Supplementary Information [file 41467_2022_30470_MOESM2_ESM.pdf]

List of supporting information:

1. Source Data files: two documents: one Excel document for raw data from each figure; one PDF document for uncropped blots.

2. Supplementary Information file: Supplementary Figures and uncropped blots presented in Supplementary Figures.

3. Supplementary Data: Mitochondrial proteins identified in IRAKM proteomic analysis.
