# Supplementary material for: IL-1R-IRAKM-Slc25a1 signaling axis reprograms lipogenesis in adipocytes to promote diet-induced obesity in mice
Source: Nat Commun. 2022 May 18;13:2748. doi: 10.1038/s41467-022-30470-w (PMC9117277; doi:10.1038/s41467-022-30470-w)
Supplement: Supplementary file 4 — Reporting Summary [file 41467_2022_30470_MOESM4_ESM.pdf]

nature research

Corresponding author(s): XIao Li

Last updated by author(s): Apr 8, 2022

Reporting Summary

Nature Research wishes to improve the reproducibility of the work that we publish. This form provides structure for consistency and transparency in reporting. For further information on Nature Research policies, see our Editorial Policies and the Editorial Policy Checklist.

Statistics

For all statistical analyses, confirm that the following items are present in the figure legend, table legend, main text, or Methods section.

n/a	Confirmed	
☐	☒	The exact sample size (*n*) for each experimental group/condition, given as a discrete number and unit of measurement
☐	☒	A statement on whether measurements were taken from distinct samples or whether the same sample was measured repeatedly
☐	☒	The statistical test(s) used AND whether they are one- or two-sided<br>*Only common tests should be described solely by name; describe more complex techniques in the Methods section.*
☐	☒	A description of all covariates tested
☐	☒	A description of any assumptions or corrections, such as tests of normality and adjustment for multiple comparisons
☐	☒	A full description of the statistical parameters including central tendency (e.g. means) or other basic estimates (e.g. regression coefficient) AND variation (e.g. standard deviation) or associated estimates of uncertainty (e.g. confidence intervals)
☐	☒	For null hypothesis testing, the test statistic (e.g. *F*, *t*, *r*) with confidence intervals, effect sizes, degrees of freedom and *P* value noted<br>*Give P values as exact values whenever suitable.*
☒	☐	For Bayesian analysis, information on the choice of priors and Markov chain Monte Carlo settings
☒	☐	For hierarchical and complex designs, identification of the appropriate level for tests and full reporting of outcomes
☒	☐	Estimates of effect sizes (e.g. Cohen's *d*, Pearson's *r*), indicating how they were calculated

Our web collection on statistics for biologists contains articles on many of the points above.

Software and code

Policy information about availability of computer code

Data collection	No software was used for data collection.
Data analysis	All the image quantification analyses were performed using ImageJ software version 1.53i 24. GraphPad Prism 8 software was used for all graphical and statistical analysis.

For manuscripts utilizing custom algorithms or software that are central to the research but not yet described in published literature, software must be made available to editors and reviewers. We strongly encourage code deposition in a community repository (e.g. GitHub). See the Nature Research guidelines for submitting code & software for further information.

Data

Policy information about availability of data

All manuscripts must include a data availability statement. This statement should provide the following information, where applicable:

- Accession codes, unique identifiers, or web links for publicly available datasets
- A list of figures that have associated raw data
- A description of any restrictions on data availability

The authors declare that all data supporting this study’s findings are available within this paper and its Supplementary and Source Files. The mass spectrometry proteomics data have been deposited to the ProteomeXchange Consortium via the PRIDE partner repository with the dataset identifier PXD031934 (Hyperlink:https://www.ebi.ac.uk/pride/archive/projects/PXD031934/private). All datasets generated during and/or analyzed during the current study are also available from the corresponding authors on reasonable request. Source data are provided with this paper.

Field-specific reporting

Please select the one below that is the best fit for your research. If you are not sure, read the appropriate sections before making your selection.

☒ Life sciences ☐ Behavioural & social sciences ☐ Ecological, evolutionary & environmental sciences

For a reference copy of the document with all sections, see nature.com/documents/nr-reporting-summary-flat.pdf

Life sciences study design

All studies must disclose on these points even when the disclosure is negative.

Sample size	The sample size was chosen to assure significant statistical differences and reproducibility of the results. Also, the maximum number of available mice for each experiment was used respecting the guidelines of animal welfare. At least, six mice were used in each experiment. For the experiments performed with cells, no sample size calculation was performed and sample size was determined based on variability across independent experiments.
Data exclusions	No data was excluded.
Replication	Every experiment shown as repeated at least three times as indicated in the figure legends. All of replicate experiments produced consistent, statistically significant data, as indicated in figure legends.<br>For quantification of histological data, 3 views per slide, 3 sections per mouse (n=5) were included in the analysis.
Randomization	To prevent bias, analyzed samples did not bear any information about the mouse genotype, diet or treatment performed. Analyzes were done in a blinded manner without taking into account to which experimental groups the data belong to. Only age-matched mice were used for the study. Littermates of the same sex were randomly assigned to either experimental or control groups (described in Methods). Randomization is not relevant to cell culture-based experiments. The same number of cells were used for the experiments and the experiments were well-controlled.
Blinding	To reduce potential experimental bias, the investigators were blinded to group allocation during data collection and/or analysis for H&E staining and Mac2 staining. While blinding to group allocation was not always possible due to COVID19 restrictions, the data collection and analysis were performed carefully by at least two different investigators.

Reporting for specific materials, systems and methods

We require information from authors about some types of materials, experimental systems and methods used in many studies. Here, indicate whether each material, system or method listed is relevant to your study. If you are not sure if a list item applies to your research, read the appropriate section before selecting a response.

Materials & experimental systems

n/a	Involved in the study	
☐	☒	Antibodies
☐	☒	Eukaryotic cell lines
☒	☐	Palaeontology and archaeology
☐	☒	Animals and other organisms
☒	☐	Human research participants
☒	☐	Clinical data
☒	☐	Dual use research of concern

Methods

n/a	Involved in the study	
☒	☐	ChIP-seq
☒	☐	Flow cytometry
☒	☐	MRI-based neuroimaging

Antibodies

Antibodies used	Antibodies used for immunoblotting, and immunohistochemistry were as follows: rabbit polyclonal anti-Slc25a1 (1μg for IP, 1:1,000 for WB) (15235-1-AP) and mouse monoclonal Slc25a1 (1:1,000) (66771-1-Ig; Clone: 1F11A3) were purchased from Proteintech. Goat polyclonal anti-IRAKM (1μg for IP, 1:1,000 for WB) (PAB7483) was obtained from Abnova. Rabbit polyclonal anti-Pgc1α (1μg for IP, 1:1,000 for WB) (AB3242) was purchased from Millipore. Mouse monoclonal anti-Ucp1 (1:1,000) (MAB6158; Clone: 536435) was obtained from R&D Systems. Rabbit polyclonal anti-IRAK2 (1:1,000) (ab62419), Mouse monoclonal anti-Cytochrome C reductase (1:1,000) (ab110252; Clone: 16D10AD9AH5), and Mouse monoclonal anti-Mac2 (1:50 for IHC) (ab2785; Clone: A3A12) were purchased from Abcam. Mouse monoclonal anti-Actin (1:1,000) (sc-8432; Clone: C-2), Mouse monoclonal anti-VDAC (1:1,000) (sc-390996; Clone: B-6), Mouse monoclonal anti-Tubulin (1:1,000) (sc-166729; Clone: F-1), and Mouse monoclonal anti-Tom20 (1:1,000) (sc-17764; Clone: F-10) were obtained from Santa Cruz Biotechnology. Rabbit monoclonal anti-IRAK1 (1:1,000) (4504; Clone: D51G7), Rabbit polyclonal anti-IRAK4 (1:1,000) (4363), Rabbit monoclonal anti-MyD88 (1:1,000) (4283; Clone: D80F5), Mouse monoclonal anti-Diablo (1:1,000) (2954; Clone: 79-1-83), Rabbit polyclonal anti-Hsp60 (1:1,000) (4870), Rabbit monoclonal anti-Flag (1:1,000) (14793; Clone: D6W5B), Rabbit monoclonal anti-His (1:1,000) (12698; Clone: D3I1O), Rabbit monoclonal anti-GST (1:1,000) (2625; Clone: 91G1), Mouse monoclonal anti-Phospho-Threonine (1:1,000) (9386; Clone: 42H4), and Rabbit polyclonal anti-Acetylated-Lysine (1:1,000) (9441) were purchased from Cell Signaling Technology.

Validation	All antibodies used here were validated by the manufacture. Validation statements and literature citations are available on the manufacturer's websites.<br>rabbit polyclonal anti-Slc25a1: https://www.ptglab.com/products/SLC25A1-Antibody-15235-1-AP.htm<br>mouse monoclonal Slc25a1: https://www.ptglab.com/products/SLC25A1-Antibody-66771-1-Ig.htm<br>Goat polyclonal anti-IRAKM: http://www.abnova.com/products/products_detail.asp?catalog_id=PAB7483<br>Rabbit polyclonal anti-Pgc1α: https://www.emdmillipore.com/US/en/product/Anti-PGC-1-Antibody,MM_NF-AB3242<br>Mouse monoclonal anti-Ucp1: https://www.rndsystems.com/products/human-mouse-ucp1-antibody-536435_mab6158<br>Rabbit polyclonal anti-IRAK2: https://www.abcam.com/irak2-antibody-ab62419.html<br>Mouse monoclonal anti-Cytochrome C reductase: https://www.abcam.com/ubiquinol-cytochrome-c-reductase-core-protein-i-antibody-16d10ad9ah5-ab110252.html<br>Mouse monoclonal anti-Mac2: https://www.abcam.com/galectin-3-antibody-a3a12-ab2785.html<br>Mouse monoclonal anti-Actin: https://www.scbt.com/p/actin-antibody-c-2<br>Mouse monoclonal anti-VDAC: https://www.scbt.com/p/vdac1-antibody-b-6?requestFrom=search<br>Mouse monoclonal anti-Tubulin: https://www.scbt.com/p/beta-tubulin-antibody-f-1?requestFrom=search<br>Mouse monoclonal anti-Tom20: https://www.scbt.com/p/tom20-antibody-f-10?requestFrom=search<br>Rabbit monoclonal anti-IRAK1: https://www.cellsignal.com/products/primary-antibodies/irak1-d51g7-rabbit-mab/4504<br>Rabbit polyclonal anti-IRAK4: https://www.cellsignal.com/products/primary-antibodies/irak4-antibody/4363<br>Rabbit monoclonal anti-MyD88: https://www.cellsignal.com/products/primary-antibodies/myd88-d80f5-rabbit-mab/4283<br>Mouse monoclonal anti-Diablo: https://www.cellsignal.com/products/primary-antibodies/smac-diablo-79-1-83-mouse-mab/2954<br>Rabbit polyclonal anti-Hsp60: https://www.cellsignal.com/products/primary-antibodies/hsp60-d307-antibody/4870<br>Rabbit monoclonal anti-Flag: https://www.cellsignal.com/products/primary-antibodies/dykddddk-tag-d6w5b-rabbit-mab-binds-to-same-epitope-as-sigma-s-anti-flag-m2-antibody/14793<br>Rabbit monoclonal anti-His: https://www.cellsignal.com/products/primary-antibodies/his-tag-d3i1o-xp-rabbit-mab/12698<br>Rabbit monoclonal anti-GST: https://www.cellsignal.com/products/primary-antibodies/gst-tag-91g1-rabbit-mab/2625<br>Mouse monoclonal anti-Phospho-Threonine: https://www.cellsignal.com/products/primary-antibodies/phospho-threonine-42h4-mouse-mab/9386<br>Rabbit polyclonal anti-Acetylated-Lysine: https://www.cellsignal.com/products/primary-antibodies/acetylated-lysine-antibody/9441

Eukaryotic cell lines

Policy information about cell lines

Cell line source(s)	293T cells (ATCC® CRL-3216™) were purchased from ATCC.
Authentication	The cell line was not authenticated
Mycoplasma contamination	Cells tested negative for mycoplasm contamination
Commonly misidentified lines<br>(See ICLAC register)	No commonly misidentified cell lines were used in the study.

Animals and other organisms

Policy information about studies involving animals; ARRIVE guidelines recommended for reporting animal research

Laboratory animals	Only adult male mice, aged 6-8 weeks were used for this study. The mice were bred in a specific pathogen-free environment at a temperature of 21°C, relative humidity of 50-70% and under a constant 12-h light/dark cycle. IRAKM knockout mice were generated by Dr. Richard Flavell (Yale School of Medicine, New Haven). IRAKM flox/flox mice were generated by our lab. Adipocyte specific deletion of IRAKM (IRAKMAKO) was obtained by breeding IRAKM flox/flox mice with Adiponectin-Cre transgenic mice (Jackson Laboratory, 028020). IRAKM kinase-dead(K205A) knock-in mice were generated by CRISPR/Cas-mediated genome engineering (Cyagen Biosciences).
Wild animals	This study did not involve wild animals.
Field-collected samples	This study did not involve field-collected samples.
Ethics oversight	All procedures using animals were approved by the Cleveland Clinic Institutional Animal Care and Use Committee.

Note that full information on the approval of the study protocol must also be provided in the manuscript.
