# Supplementary material for: IL-1R-IRAKM-Slc25a1 signaling axis reprograms lipogenesis in adipocytes to promote diet-induced obesity in mice
Source: Nat Commun. 2022 May 18;13:2748. doi: 10.1038/s41467-022-30470-w (PMC9117277; doi:10.1038/s41467-022-30470-w)

Fig. 1a

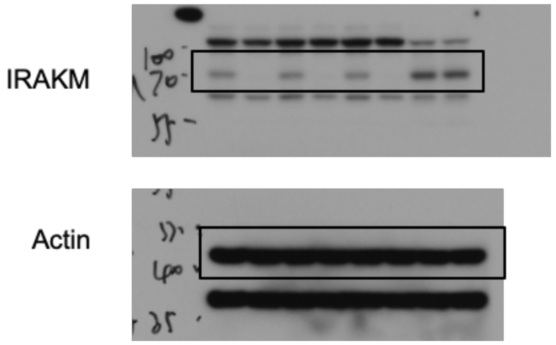

Fig. 2I

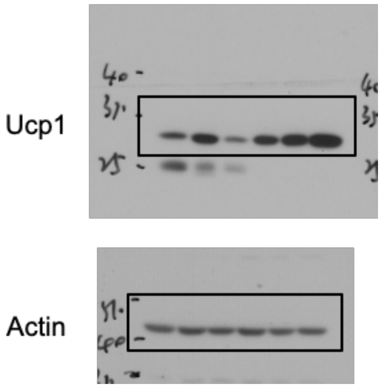

Fig. 3d

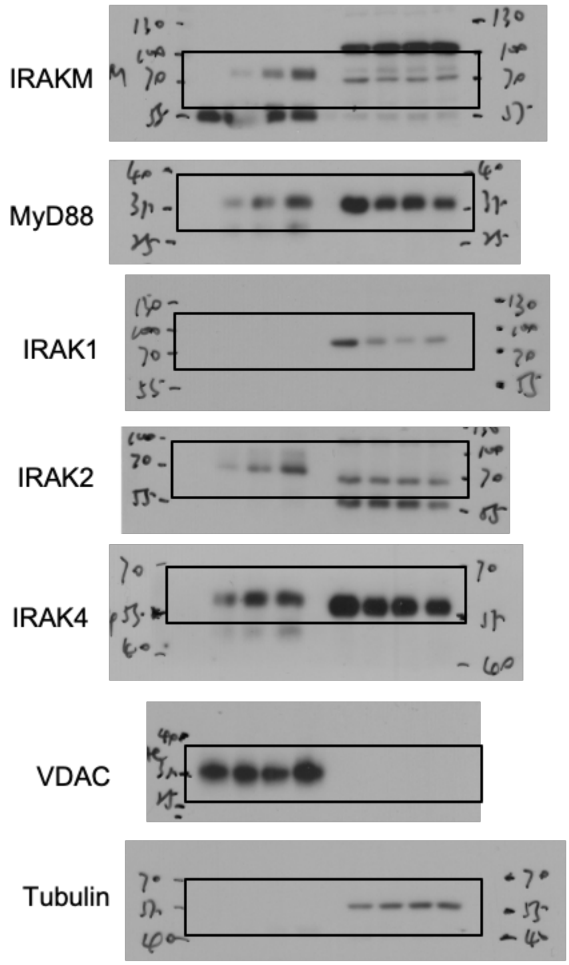

Fig. 3e

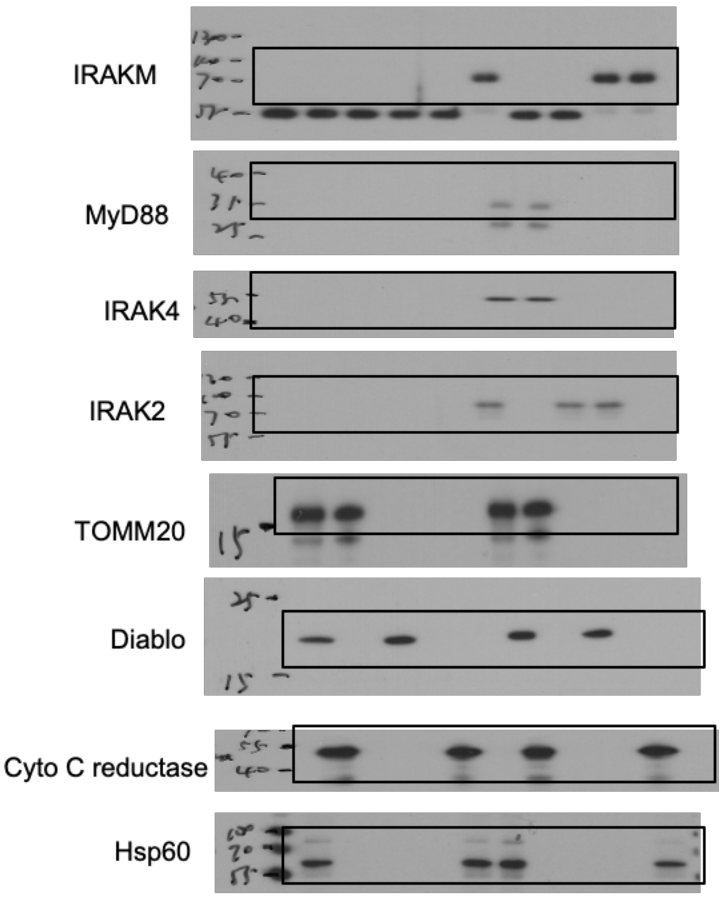

Fig. 3h

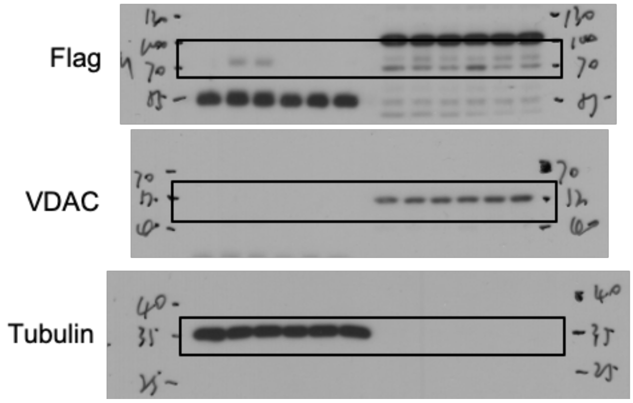

Fig. 4a

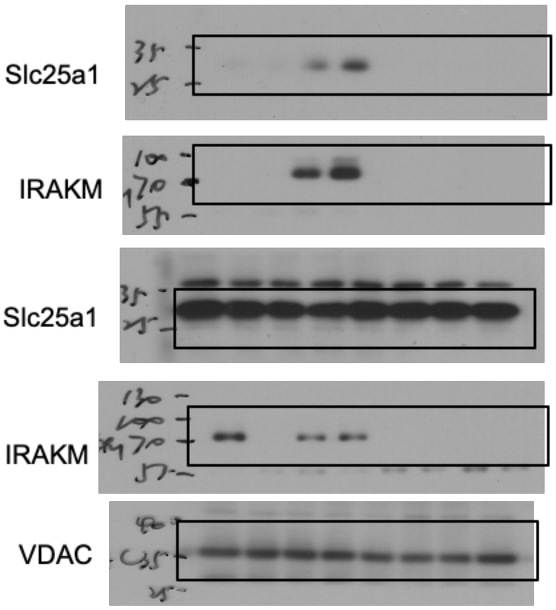

Fig. 4b

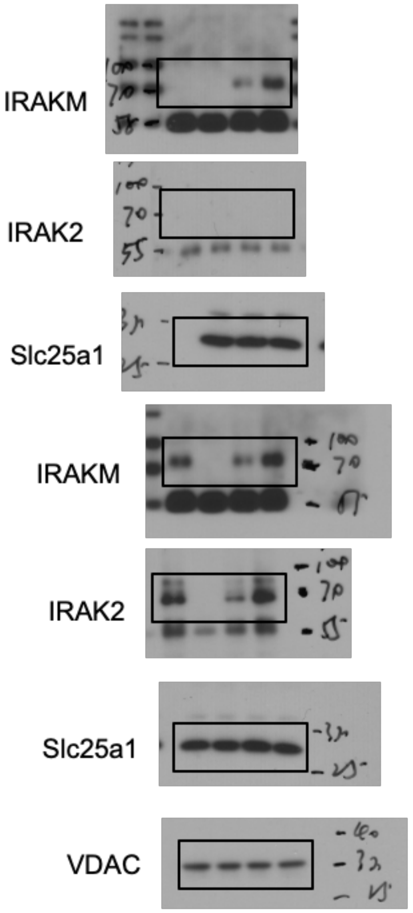

Fig. 4e

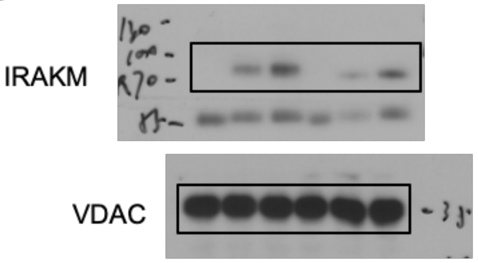

Fig. 4f

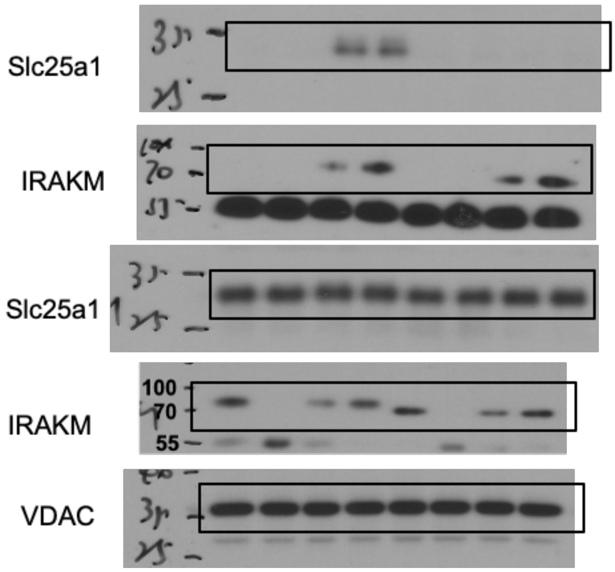

Fig. 4i

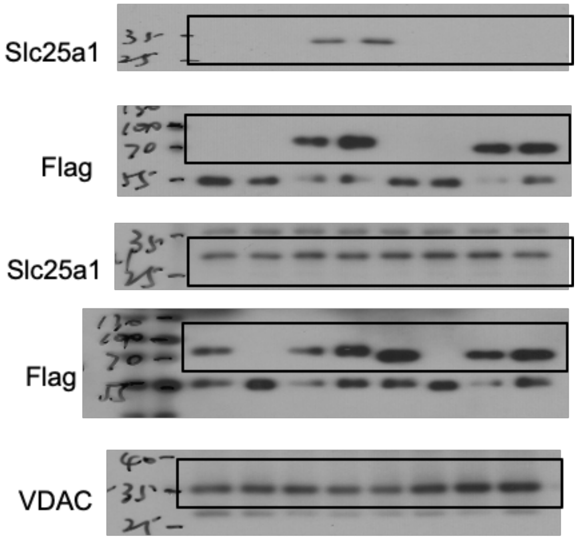

Fig. 5a

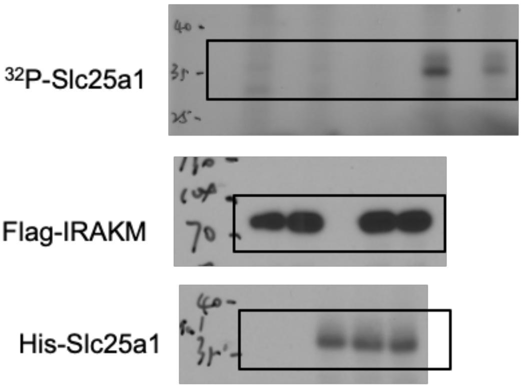

Fig. 5c

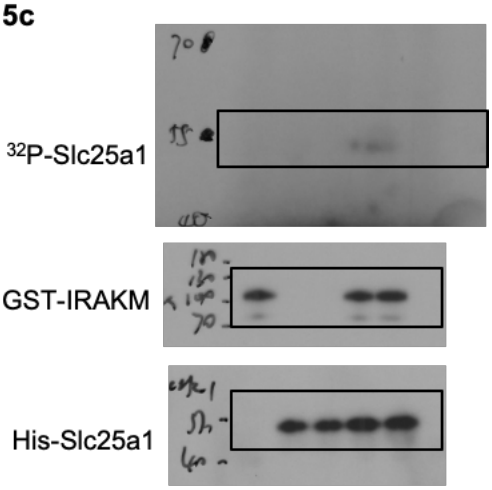

Fig. 5d

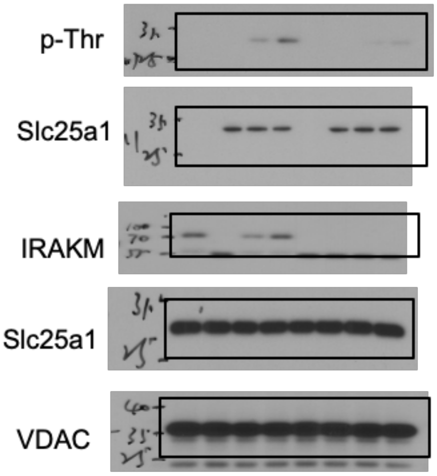

Fig. 5e

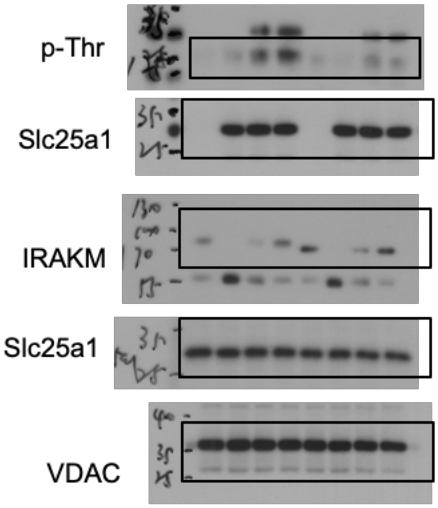

Fig. 5f

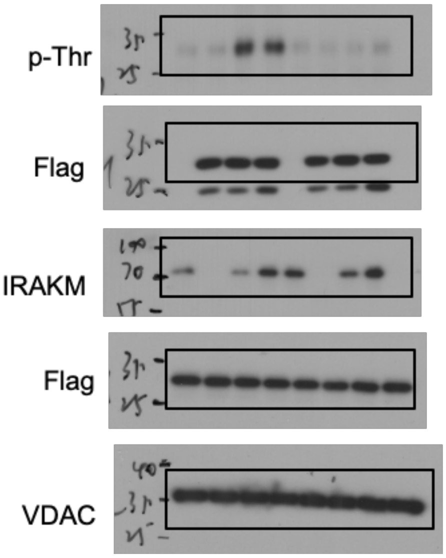

Fig. 6a

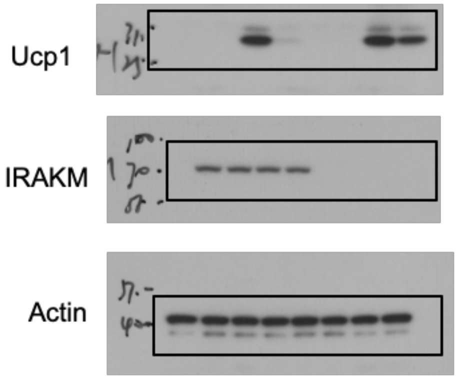

Fig. 6b

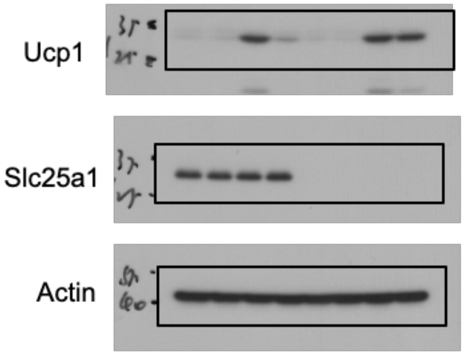

Fig. 6c

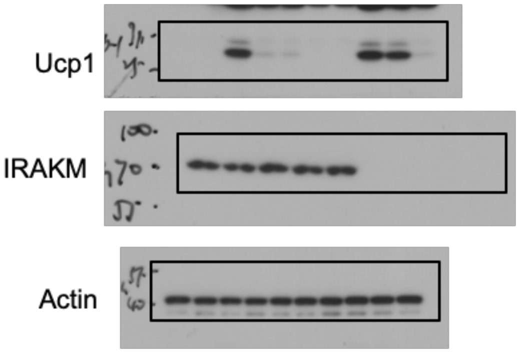

Fig. 6f

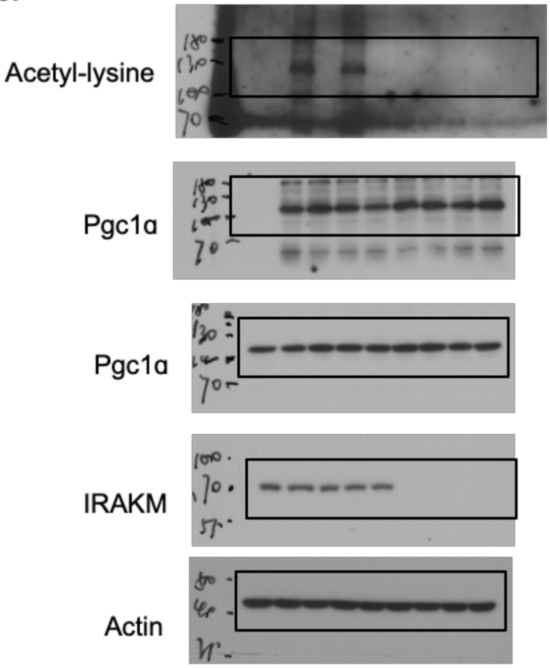

Fig. 6g

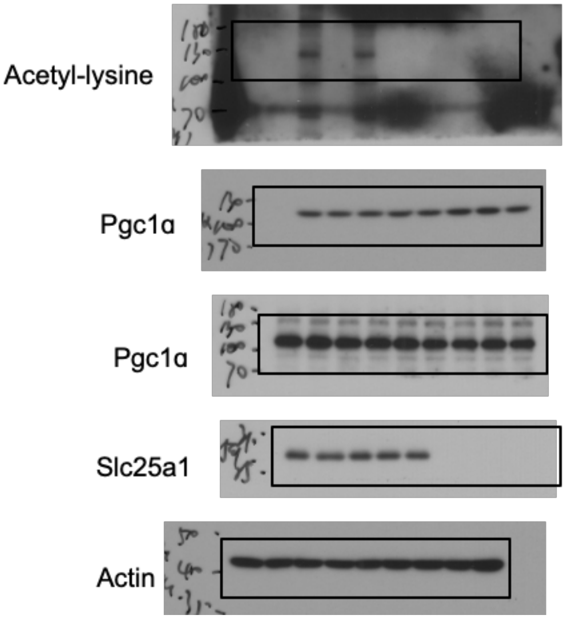

Fig. 7k

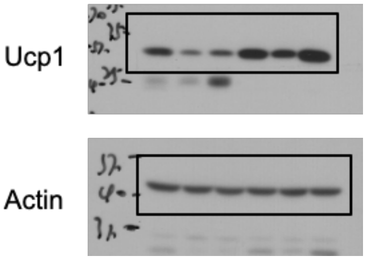

Supplement: Supplementary file 5 — Source Data [file 41467_2022_30470_MOESM5_ESM.zip › Source Data /Uncropped blots.pdf]
